# Inflammation and emotion regulation: a narrative review of evidence and mechanisms in emotion dysregulation disorders

**DOI:** 10.1042/NS20220077

**Published:** 2023-11-15

**Authors:** Flavia Petruso, Alexis E. Giff, Beatrice A. Milano, Maurilio Menduni De Rossi, Luigi Francesco Saccaro

**Affiliations:** 1 Politecnico of Milan, Milan, Italy; 2 Department of Neuroscience, School of Life Sciences, École Polytechnique Fédérale de Lausanne, Switzerland; 3 Sant’Anna School of Advanced Studies, Pisa, Italy; 4 University of Pisa, Pisa, Italy; 5 Department of Psychiatry, Faculty of Medicine, University of Geneva, Switzerland; 6 Department of Psychiatry, Geneva University Hospital, Switzerland

**Keywords:** Attention Deficit/Hyperactivity Disorder, Bipolar Disorder, Borderline Personality Disorder, emotion regulation, inflammation, neuroinflammation

## Abstract

Emotion dysregulation (ED) describes a difficulty with the modulation of which emotions are felt, as well as when and how these emotions are experienced or expressed. It is a focal overarching symptom in many severe and prevalent neuropsychiatric diseases, including bipolar disorders (BD), attention deficit/hyperactivity disorder (ADHD), and borderline personality disorder (BPD). In all these disorders, ED can manifest through symptoms of depression, anxiety, or affective lability. Considering the many symptomatic similarities between BD, ADHD, and BPD, a transdiagnostic approach is a promising lens of investigation. Mounting evidence supports the role of peripheral inflammatory markers and stress in the multifactorial aetiology and physiopathology of BD, ADHD, and BPD. Of note, neural circuits that regulate emotions appear particularly vulnerable to inflammatory insults and peripheral inflammation, which can impact the neuroimmune milieu of the central nervous system. Thus far, few studies have examined the link between ED and inflammation in BD, ADHD, and BPD. To our knowledge, no specific work has provided a critical comparison of the results from these disorders. To fill this gap in the literature, we review the known associations and mechanisms linking ED and inflammation in general, and clinically, in BD, ADHD, and BD. Our narrative review begins with an examination of the routes linking ED and inflammation, followed by a discussion of disorder-specific results accounting for methodological limitations and relevant confounding factors. Finally, we critically discuss both correspondences and discrepancies in the results and comment on potential vulnerability markers and promising therapeutic interventions.

## Emotion regulation and (neuro)inflammation in emotion dysregulation disorders

Emotion regulation (ER) is the ability to modify the onset, intensity, duration, and type of emotional response to a situation [1]. Clinically, emotion dysregulation (ED), i.e., the inability to modulate emotions, can manifest through symptoms of depression, anxiety, or affective lability, i.e., proneness to rapid shifts between emotional states [2]. ED is a crucial component of the pathophysiology and clinical presentation of numerous psychiatric disorders. In this narrative review, we discuss the involvement of ED in three common and potentially debilitating diseases, defined henceforth as emotion dysregulation disorders (EDD): bipolar disorder (BD) [3,4], attention deficit/hyperactivity disorder (ADHD) [5], and borderline personality disorder (BPD) [6,7]. These diseases have a profound socioeconomic impact and high prevalence (0.8–2.4% for BD [8,9], 2.5–15% for ADHD [10], and 2.7–6% for BPD [11,12], see also [13]). Furthermore, they are often comorbid with one another [14–25], and previous literature highlighted their substantial similarities in terms of symptoms and psychopathology [14–27]. While certain aspects of these three highly prevalent disorders might be found in other psychiatric conditions, they share a distinct set of common features that accentuate the significance of investigating ED within these clinical populations. Indeed, most of their shared symptoms, such as anxiety, impulsivity, externalizing behaviours or proneness to substance abuse, could potentially arise from underlying ED [23]. Furthermore, the onset or early prodromal signs of these disorders typically occur during adolescence and young adulthood [28,29]; considering that ER circuits typically mature in the early twenties [30,31], this temporal alignment suggests that exploring ED-associated vulnerability markers in EDD could hold considerable clinical implications. Indeed, increasing evidence shows substantial benefits of early interventions in individuals at risk for EDD [32–36], but there is a compelling need for deeper pathophysiological insights. Thus, we endeavour to highlight the importance of investigating ED in these diseases both from a prognostic and diagnostic viewpoint, as well as for preventive and therapeutic interventions.

Since the EDD we focus on also involve mood symptoms, it is important to emphasize the distinction between emotion and mood. Both play integral roles in our everyday behaviour and overall mental health but are distinct regarding timespan, intensity, triggers, and associated expressions, among other features. One possible definition is that emotions are relatively intense and short-lived psychological experiences that are typically triggered by identifiable events and accompanied by universal facial and behavioural expressions (e.g., anger, joy…); meanwhile, mood is the less acute background state (e.g., depression, euthymia…) that may fluctuate but usually persist for much longer, is not always triggered by specific events, and does not necessarily have unique expression cues [37]. Emotions may be congruent or discordant with mood states (e.g., a person whose mood is sad may still display a temporary positive emotion, for instance, smiling out of joy when seeing someone they love). ED refers to difficulties with modulating specific emotional reactions, while mood dysregulation refers to difficulties with regulating the broader underlying mood states. While some overlap might exist between ED and mood dysregulation, a table specifying the differences between emotion and mood dysregulation for each EDD covered in this review is highlighted above (Table 1).

**Table 1:** Main features of mood and emotion dysregulation in bipolar disorder (BD), borderline personality disorder (BPD), and attention deficit/hyperactivity disorder (ADHD)

Diagnosis	Mood dysregulation symptoms	Emotion dysregulation symptoms
BD	Mood swings are a defining feature of BD, including depressive, manic, and hypomanic episodes typically lasting days, weeks, or months. Mood episodes typically last longer and are more intense than changes in mood in healthy subjects.	Difficulties in regulating and handling emotions, irrespective of the mood state. ED manifestations may vary depending on the mood state, typically showing a lower effect size during euthymic states [161]. Excessive focus on negative life events, increased ruminations, greater risky or impulsive behaviours in response to emotions, and inhibition of ongoing emotion-expressive behaviour [161].
BPD	More rapid changes in mood than in BD, prone to significant decreases in overall positive mood, and decreased mood labeling [360]. Mood states can even change within a single day. Mood changes may often be prompted by external events or perceived threats to the person's self-image or relationships.	Heightened emotional sensitivity and lability is a defining feature of BPD. Negativity bias in emotion recognition, lowered emotional granularity and emotional labeling. Negative emotionality is also common [360]. Emotional states can become extremely intense and manifest with impulsive actions, self-harm, or suicide.
ADHD	Rapid and unpredictable changes in mood [322]. Mood component is less prominent than in BD and BPD.	Higher irritability and temper outbursts, increased tendency to become angry, disagree, or be critical of others, prominent negative emotionality [322]. ED may manifest with impulsivity, externalizing behaviours, or substance abuse, but is less prominent than in BPD.

While emotion dysregulation (ED) and mood dysregulation are present in all three of these disorders, they can express through slightly different manifestations, as detailed in the table.

Deficient or maladaptive ER has a strong neurobiological equivalent in these disorders, with documented brain alterations spanning multiple ER-associated circuits, including impairments in the functional and structural connectivity of limbic ER networks [38–44]. Regions involved in ER that often exhibit alterations in EDD include the amygdala, the hippocampus, the dorsolateral (dlPFC), dorsomedial (dmPFC), ventrolateral (vlPFC), and ventromedial (vmPFC) prefrontal cortex, the anterior cingulate cortex (ACC), the inferior (IFG) and superior (SFG) frontal gyrus, alongside parietal and temporal regions, and the insula [45–49]. Considering the shared features in both neurobiological and clinical spheres, EDD may be especially suitable for examination through a novel approach that overcomes traditional diagnostic boundaries and highlights the similarities between the disorders, i.e., the transdiagnostic approach [50]. It emphasises the importance of including multiple measures (e.g., circuit-level, behavioural, psychological) in research designs to examine constructs that span different psychiatric diagnoses with overarching features and symptoms, leading to an integrative rather than reductionistic framework. Interestingly, ED has been proposed as a sixth domain in the RdoC, a transdiagnostic framework introduced by the National Institutes of Mental Health [51].

**Figure 1:**
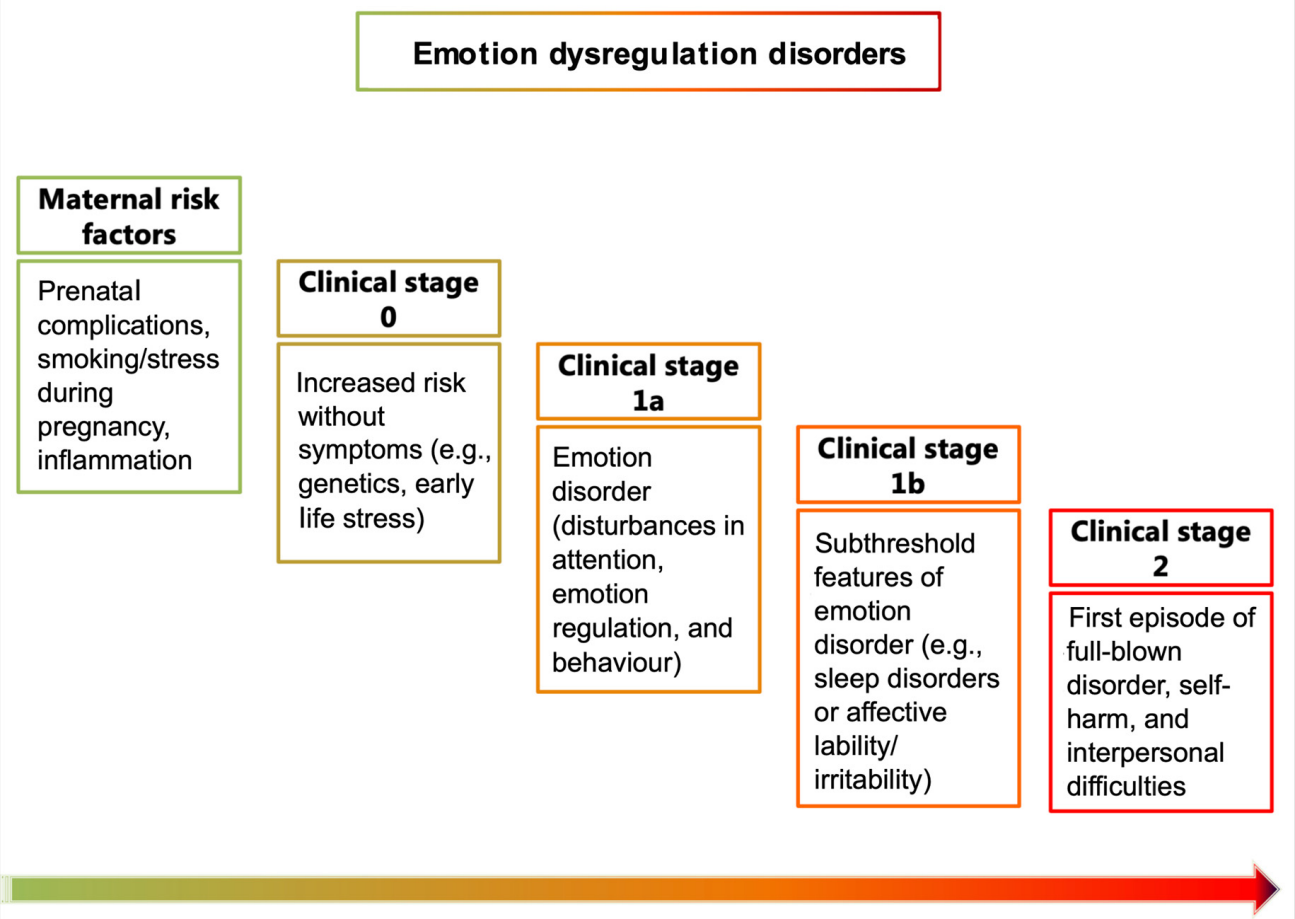
Proposed clinical staging model of emotion dysregulation Starting with potential maternal risk factors, emotion dysregulation (ED) may present with the following stages: 0. Increased risk with no specific symptoms, 1a. Mild or nonspecific symptoms, 1b. Subthreshold features, 2. First episode of full-blown disorder.

**Figure 2:**
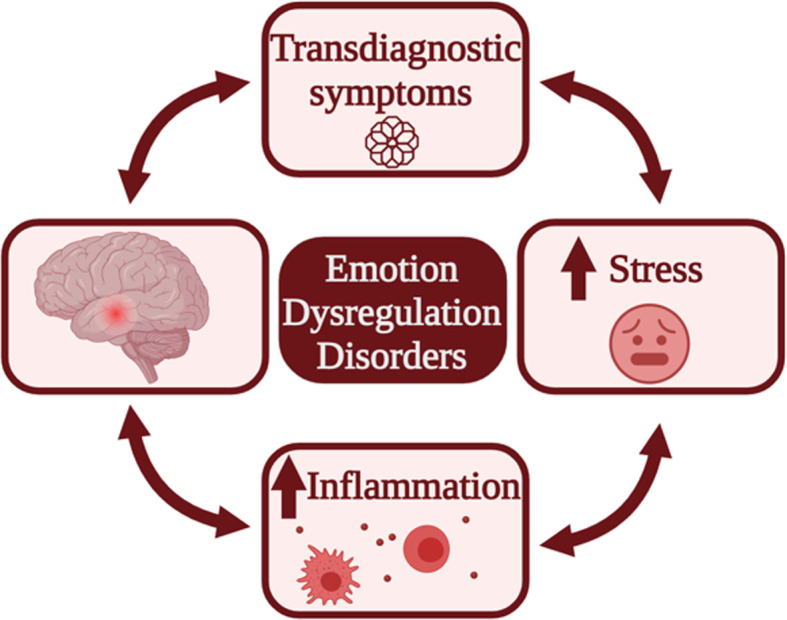
Theoretical framework recapitulating the links between inflammation, stress, emotion regulation networks, and clinical symptoms (adapted from [250]) Emotion dysregulation (ED) symptoms may lead to higher chronic stress and trauma, which are in turn associated with a proinflammatory state. Neurotoxic effects of inflammation can negatively impact the neural circuits of emotion regulation (ER), exacerbating symptoms. Thus, although the direction of potential causality between these elements remains to be ascertained, it appears likely that a vicious cycle perpetuating inflammation, central nervous system (CNS) dysfunction, chronic stress, and ED symptoms may exist. Upward red arrows indicate increases.

Numerous findings on the multifactorial aetiology of EDD [52–56] suggest that a multimodal approach is required for a more comprehensive characterisation of these disorders. Recent evidence on the physiopathology of BD [21,57–59], ADHD [60–62], and BPD [21,63] points toward an involvement of inflammation and stress and suggests that these features play a pivotal role in EDD. Symptoms of ED can expose subjects to severe chronic stress. Moreover, early life stress (ELS), i.e., exposure to trauma, abuse, or events perceived as highly stressful during the neurodevelopmental period, contributes to the pathogenesis and maintenance of EDD as displayed in Figure 1 (see also [17,64]). Therefore, an association between inflammation, stress and ED has been hypothesized, while also being supported by results on different biological scales. As defined by the World Health Organization, stress is ‘a state of worry or mental tension’ that can precede various health problems [65]. Common classifications include acute stress, chronic stress, and stress induced by early life adversities (i.e., ELS). As ADHD, BPD, and BD diagnosis or prodromal symptoms frequently emerge during the developmental period and ER circuits continue to develop until the early twenties [28,29], this study will primarily, though not exclusively, focus on ELS. Stress itself is linked with a proinflammatory status via modulation of the hypothalamic–pituitary–adrenal (HPA) axis [21,60,66,67] and a selective vulnerability of limbic ER circuits to peripheral inflammation has been consistently documented [68–70], which may play a crucial role in EDD [68,69,71]. For instance, in BD patients, inflammatory biomarkers have been identified in ER-associated brain regions, such as the hippocampus [72]. Recent cross-sectional evidence from ADHD and BPD has also revealed that a history of childhood maltreatment, which has been associated with increased peripheral inflammation in humans [73–76], is linked to maladaptive emotional responses in adulthood [77]. A vicious cycle may therefore exist in EDD, in which stress and the associated proinflammatory state disrupt limbic connectivity, exacerbating ED symptoms and exposing patients to further stress and immune dysregulation. This ultimately perpetuates a cycle simplified in Figure 2. Several mechanistic hypotheses have been proposed to explain how peripheral inflammation can translate into central inflammation, neuronal damage, or other alterations in the central nervous system (CNS), as discussed in the next sections. This emerging evidence suggests that peripheral inflammation may affect limbic circuits in EDD patients and further supports the need for a multimodal approach when studying these disorders.

However, to the best of our knowledge, there are no literature reviews associating ED in BPD with inflammation, stress, or neuroimaging markers, and just a few explore only some of these associations in ADHD [60,78–80] and BD [21,57,81–83]. This narrative review aims to fill this literature gap by examining evidence of the connection between ED and inflammation (alongside the associated physiopathological processes) in EDD, which may represent a promising vulnerability marker to guide early interventions in at-risk individuals. First, in the next section, we report findings associating ED and inflammation, presenting their physiological correlates and potential mechanisms from human or animal studies. Then, in the following sections, we present results on the associations between inflammatory traits or markers and clinical or neurobiological correlates of ED in BD, ADHD, and BPD patients. Finally, we critically review and discuss the results presented in this work, highlighting the clinical implications, the limitations, and possible future directions of investigation.

## Emotion regulation and (neuro)inflammation: clinical and experimental evidence on associations and potential mechanisms

### Clinical evidence links peripheral inflammation and emotion dysregulation

As mentioned in the previous section, one possible mediator of the negative health outcomes associated with ED is inflammation. Multiple studies have investigated the correlation between ED symptoms and peripheral inflammation markers to explore this hypothesis. Clinically, ER can be measured through self-assessment questionnaires, including the Emotion Regulation Questionnaire (ERQ) [84] or the Difficulties in Emotion Regulation Scale (DERS) questionnaire [85]. Both questionnaires measure the deployment of behaviours aimed at controlling emotional responses, known as ER strategies, which have been linked to different physical and mental well-being outcomes [49,78,86]. The most investigated strategies are cognitive reappraisal and expressive suppression. Cognitive reappraisal is an *antecedent-focused* strategy where the individual positively reframes the interpretation of situations before their occurrence [1,87] it is regarded as adaptive in most contexts [88–91]. Conversely, expressive suppression is a *response-focused* strategy where a subject voluntarily blocks the expression of a negative emotion after experiencing it; it is usually classified as less adaptive or even maladaptive, having been tied to poorer executive functions [88] and negative health outcomes [49] such as increased cardiovascular risk [89]. One intriguing possibility is that inflammation mediates the link between ED and adverse health outcomes; converging evidence suggests that different ER strategies may be associated with specific inflammatory markers [92–98], as reported in Table 2.

**Table 2:** Main articles linking peripheral inflammation with emotion regulation

	Main findings	Sample	Measure(s) of ER/ED or brain correlates	Measure(s) to evaluate inflammation	Study type	Covariates	Directionality of the findings
**Articles linking peripheral inflammation with emotion regulation strategies and behaviours**							
Moriarity et al., 2023* [92]	Positive link between emotion dysregulation and increased peripheral inflammation	Final number of articles = 38	Heterogeneous measures of ER, including ERQ and DERS	Peripheral inflammatory markers	Systematic review	Varying with the study	Emotion regulation broadly associated with higher peripheral concentrations of proinflammatory proteins.
Appleton et al., 2013 [93]	Higher reappraisal scores were negatively associated with peripheral levels of CRP, higher suppression scores were associated with higher peripheral levels of CRP	379 healthy adults; mean age = 42.2 years, SD = 1.7 years	ERQ for self-reported use of ER strategy (expressive suppression and cognitive reappraisal)	Peripheral CRP	Cross-sectional	Age, sex, BMI, education attainment, smoking status, ethnicity, depressive symptoms, and original study location. Child measures acquired retrospectively: being born small for gestational age, BMI, IQ, socioeconomic status, and physical health status	↑ suppression ↑ CRP, ↑ reappraisal ↓ CRP
Brown et al., 2020 [94]	Infected participants and those developing a cold after rhinovirus exposure showed a negative correlation between cognitive reappraisal and local nasal inflammatory cytokines. No correlation was found between cognitive reappraisal and cold symptoms. Subjects adopting expressive suppression did not show an increased level of nasal IL-β, IL-6, or IL-8 after rhinovirus infection. No correlation was found between expressive suppression and symptoms	212 adults, of whom 159 were infected and 63 met clinical criteria for the cold; mean age = 30, SD = 11.09 years	ERQ for self-reported use of ER strategy (expressive suppression and cognitive reappraisal)	IL-6, IL-8, and IL-1β from a nasal wash fluid sample	Experimental	Age, sex, average BMI across the sessions, education attainment, season and day of the trial	↑ reappraisal ↓ nasal inflammatory cytokines in infected patients and the ones developing a cold after exposition to pathogen, no correlation between suppression and local inflammatory cytokines in infected patients, no correlation between reappraisal/suppression and symptoms
Ellis et al., 2019 [95]	Association between ER strategies and the dysregulation of different biological systems was assessed through an AL measure. Higher expressive suppression was indirectly associated with higher AL, higher reappraisal scores were indirectly associated with lower AL. These effects were mediated by sleep quality and perceived stress	1255 adults; mean age = 54.52 years, SD = 11.71 years	ERQ for self-reported use of ER strategy (expressive suppression and cognitive reappraisal)	Allostatic load measured as a cumulative risk from seven different biological systems. Blood inflammatory markers: CRP, IL-6, E-selectin, fibrinogen and ICAM-1.	Cross-sectional	Age, sex, and ethnicity	↑ suppression ↑ allostatic load, ↑ reappraisal ↓ allostatic load. Association was indirect, mediated by global sleep quality and perceived stress
Khan, 2020 [96]	Higher expressive suppression but not cognitive reappraisal was significantly associated with a higher inflammatory composite including CRP, white blood cell counts, and fibrinogen blood levels	606 trauma-exposed veterans; mean age = 58.01 years, SD = 11.17 years	ERQ for self-reported use of ER strategy (expressive suppression and cognitive reappraisal)	Composite of peripheral CRP, white blood cell counts and fibrinogen blood levels	Cross-sectional	Age, sex, ethnicity, education, income, creatinine, diagnosis of PTSD, BMI, inactivity, drinking, smoking, and poor sleep	↑ suppression ↑ inflammatory composite, no relation between reappraisal and inflammatory composite
Lopez et al., 2020 [97]	A positive correlation was found between expressive suppression and an inflammatory composite, no association with cognitive reappraisal was found	99 adults that had lost their spouse no more than 3 months before the visit; mean age = 68.61, SD = 10.70 years	ERQ for self-reported use of ER strategy (expressive suppression and cognitive reappraisal)	Composite of peripheral IL-6, TNF-α, IL-17A, IL-2, and IFN-γ from *ex vivo* stimulation of leukocytes from a venous blood sample using T-cell agonists	Cross-sectional	Age, sex, BMI, education attainment, annual income, physical activity, smoking status, family income, antidepressant medications, time since spouse loss, sleep disturbance, and use of statins	↑ suppression ↑ inflammatory composite (and with IFN-γ TNF-α individually), no relation between reappraisal, and inflammatory composite or markers
Ospina et al., 2022 [98]	Participants who used more expressive suppression strategies had decreased circulating levels of IL-10, TNF-α, and ICAM-1 levels in their blood. No correlation was found between the biomarkers and cognitive reappraisal	117 healthy adults; mean age = 53.84 years, SD = 9.99 years	ERQ for self-reported use of ER strategy (expressive suppression and cognitive reappraisal)	Peripheral IL-6, IL-8, IL-10, TNF-α, CRP, E-selectin, ICAM-1, and fibrinogen blood levels	Cross-sectional	Age, sex, BMI, depressive symptoms, and total prescribed medications	↑ suppression ↓ inflammatory citokynes IL-10, TNF-α, and ICAM-1, no correlation between reappraisal and the same cytokines
**Neuroimaging findings linking peripheral inflammation with emotion regulation**							
Gianaros et al., 2014 [108]	Higher activity of the dACC during a reappraisal-related task was linked to increased peripheral IL-6 and preclinical atherosclerosis. IL-6 mediated the link between dACC activation and preclinical atherosclerosis	157 healthy adults; mean age = 42.7 years, SD = 7.3 years	fMRI reappraisal task	Peripheral IL-6, carotid artery intima-media thickness and inter-adventitial diameter	Experimental	Age, sex, ethnicity, educational attainment, smoking status, and the cardio-metabolic risk score	↑ dACC reappraisal-related activity ↑ IL-6, no FC investigated
Kraynak et al., 2019+ [109]	(i) Retrospectively reported childhood abuse covaried negatively with amygdala-vmPFC FC but no other corticolimbic connections. (ii) IL-6 covaried negatively with FC between the amygdala and vmPFC, sgACC, and pgACC. (iii) Hippocampus-vmPFC covaried significantly with IL-6. (iv) CRP levels were negatively associated with vmPFC-amygdala and vmPFC-hippocampus FC, while analysis of limbic-PFC FC did not demonstrate any significance. IL-6 statistically modulated the influence of childhood ELS on frontoamygdala FC. Path analyses demonstrated an indirect impact of ELS on amygdala-vmPFC FC mediated by IL-6	303 healthy adults; mean age = 40.30 years, SD = 6.24 years	Resting-state fMRI; Childhood adversity and other disorder related self-reported measures	Peripheral IL-6 and CRP	Cross-sectional	Age, sex, and BMI	↑ IL-6 ↓ PFC corticolimbic FC, ↑ ELS ↓ frontoamygdala connectivity with IL-6 modulating this association
Nusslock et al., 2019 (study 1) [110]	Higher inflammatory composite scores were associated with lower activation of the ERN (including the inferior frontal gyrus, middle temporal gyrus, and precentral gyrus regarding volitional ER), and no significant association with other rsFC networks was documented.	90 healthy young adults; mean age = 24.92 years, SD = 0.57 years	Resting-state fMRI	Peripheral levels of a composite of inflammatory biomarkers, i.e*.*, CRP, IL-6, IL-10, and TNF-α	Cross-sectional	Sex	↑ inflam ↓ ERN, no correlation with other rsFC networks
Nusslock et al., 2019 (study 2) [110]	Higher inflammatory composite scores were associated with lower rsFC of the ERN and the CEN (connecting dlPFC with posterior parietal cortex). Classical monocyte counts were associated with lower rsFC in ERN and CEN. No association was found for aSN and DMN (same ROIs of study 1)	Analytic sample = 82 healthy adolescents; mean age = 13.90 years, range 12–14 years	Resting-state fMRI	Peripheral CRP, IL-6, IL-10, TNF-α (the same composite measure as study 1); circulating granulocytes, lymphocytes and classical (CD14 ++/CD16-) and nonclassical (CD14 +/CD16-) monocyte count	Cross-sectional	Age, sex, and pubertal status	↑ inflam ↓ ERN and ↓ CEN
Swartz et al., 2021 [111]	TNF-α (but not IL-6 and CRP) peripheral levels were positively correlated with amygdala-left striatum FC. A negative association was found between TNF-α and right IFG-left parietal cortex FC	Analytic sample = 70 healthy adolescents; mean age = 13.60 years, SD = 1.04 years	Resting-state FC for ROIs selected for representing ERN and CEN: bilateral amygdala, vmPFC, bilateral insula and bilateral IFG/vlPFC for ERN and bilateral MFG/dlPFC for CEN	Peripheral IL-6, TNF-α and CRP	Cross-sectional	Age, sex, BMI, time of the day blood draw, length of resting state scan, mean framewise displacement for resting state scan and medication use	↑ TNF-α ↑ FC between right AMG and left striatum, ↑ TNF-α ↓ FC between right IFG and left parietal cortex
Yuan et al., 2022+ [112]	(i) CRP levels were negatively associated with vlPFC, SFG, and anterior insula activation during an implicit ER task; (ii) the severity of ELS moderated the association between CRP and left vlPFC activation; (iii) CRP levels in adolescents did not predict FC between both amygdalae and left-vlPFC; (iv) the FC between the left vlPFC and both amygdalae was mediated by the severity of ELS	Analytic sample = 83 healthy adolescents; mean age = 15.63 years, SD = 1.10 years	Structural MRI and resting-state fMRI; cumulative severity score to assess ELS	Peripheral CRP	Experimental	Association between CRP computed for age, sex, physical status, socioeconomic status, assay batch, and scanner model	↑ CRP ↓ vlPFC, SFG and anterior insula activation during ER task, no association of CRP in terms of FC, ↑ ELS ↓ FC between bilateral AMG and L-vlPFC in adolescents with higher CRP

Studies marked with ‘+’ include measures of early life stress (ELS). AL, allostatic load; aSN, anterior salience network; BMI, body mass index; CD14, cellular differentiation 14; CEN, central executive network; CRP, C-reactive protein; dACC/pgACC/sgACC, dorsal/perigenual/subgenual anterior cingulate cortex; DMN, default mode network; ELS, early life stress; ER, emotion regulation; ERN, emotion regulation network; ERQ, emotion regulation questionnaire; FC/rsFC, resting state/functional connectivity; ICAM-1, intercellular adhesion molecule 1; IFN-γ, interferon γ; IFG/SFG/MFG, inferior/medial/superior frontal gyrus; IL-1β/IL-2/IL-6/IL-8/IL-10/IL-17, interleukin-1β/2/6/8/10/17; IQ, intelligence quotient; MRI/fMRI, functional/magnetic resonance imaging; PFC/dlPFC/vmPFC/vlPFC, dorsolateral/ventromedial/ventrolateral prefrontal cortex; PTSD, post-traumatic stress disorder; ROI, regions of interest; SD, standard deviation; TNF-α, tumour necrosis factor alpha.

A recent systematic review has tried to summarize the extant literature on the potential connection between clinical or behavioural correlates of ED and inflammation, with a final number of 38 articles included in the analysis [92]. A noteworthy finding is that, in general, ED showed a more prevalent association with elevated levels of peripheral inflammatory markers. When focusing on individual ER strategies, expressive suppression was more frequently associated with an increased peripheral proinflammatory status, whereas cognitive reappraisal was more often linked to a lower concentration of inflammatory cytokines such as C-reactive protein (CRP) and interleukin-6 (IL-6). Interestingly, other less-investigated strategies, such as negative self-referential processes, which include self-criticism, negative thoughts, and rumination, have also been linked to altered peripheral inflammatory markers and cortisol responses in healthy adults [92,99–101]. Nevertheless, the number of studies analyzed for each strategy (or set of strategies) was low, and inconsistency of the findings was frequently reported by the authors, especially from studies with smaller sample sizes.

Of note, the link between ER and health outcomes may not only be mediated by inflammation but may also involve other biological systems [49]. One previous study in adults sought to assess the relationship between expressive suppression and cognitive reappraisal [95] with a general measure of allostatic load [102,103], including indexes of cumulative risk from different biological systems (i.e., cardiovascular, glucose and lipid metabolism, inflammation, sympathetic nervous system, and the HPA axis). They found that a general increase in allostatic load was indirectly associated with higher use of expressive suppression, and a decrease was tied to cognitive reappraisal, with this association being mediated by sleep quality and perceived stress [95]. Future work is warranted to elucidate the nature of these interactions; we discuss some of them later in this section.

The intrinsic limitation of these works, which may explain to some degree the partly contradictory results, lies in the cross-sectional nature of most studies [93,95–98]. The single-point administration of ER questionnaires, often temporally distant from the sample collection, may also inhibit accurate correlations. Furthermore, a rigid categorization of emotional responses as intrinsically adaptive or maladaptive may not fully capture the complexity of the problem, as the physical effects of ER strategies may depend on the context of deployment [87,104], which should be taken into consideration in future study designs.

Despite these caveats, most of the presented studies provide the groundwork to support that different ER strategies may be tied to immune alterations, compatibly with strengthening the hypothesis that inflammation is a mediator of adverse health outcomes associated with ED. It is also possible, however, that peripheral inflammation and neuroinflammation, i.e., inflammation occurring in the CNS beyond the blood–brain barrier (BBB), act as *primum movens* of ED. As discussed later in this section, both types of inflammation may cause neuronal damage through a plethora of mechanisms, and limbic circuits demonstrate a selective vulnerability to stress and inflammation [68,70,105]. Alternatively, from a neurodevelopmental perspective, early alterations of ER-associated circuits may lead to ED and inflammation. These mechanisms may further impact one another in a vicious cycle, as summarized in Figure 2. In the upcoming subsection, we present findings from neuroimaging studies linking ER and inflammation.

### Neuroimaging findings link peripheral inflammation and emotion dysregulation

**Figure 3:**
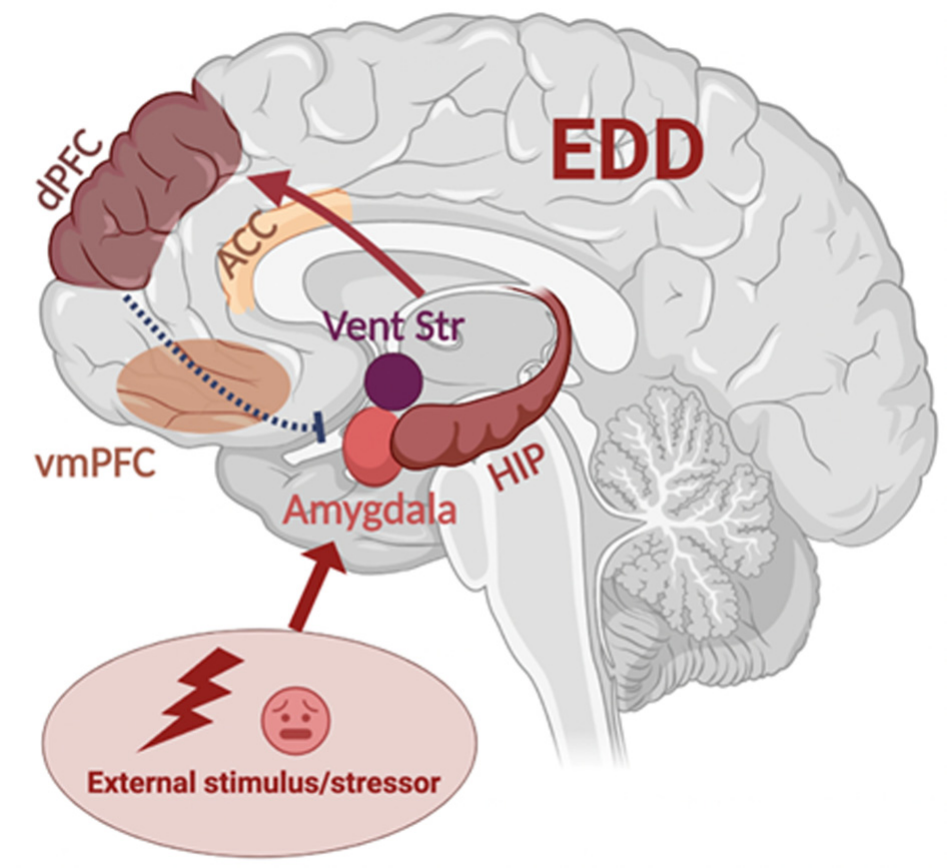
Emotion dysregulation disorders (EDD) display altered functional connectivity in the brain structures that control emotions Under the effect of stressors or external stimuli, limbic structures such as the amygdala, ventral striatum (Vent Str), and hippocampus (HIP) are activated and affect emotional reactivity. In turn, these areas send excitatory inputs (red arrows) to the dorsal prefrontal cortex (dPFC) and associated frontal areas, including the ventromedial prefrontal cortex (vmPFC) (afferent projections not shown), which project back to the limbic system with inhibitory connections (blue dashed arrows), down-regulating the emotional response. In EDD, there is evidence that this signalling is frequently altered, with abnormal, heightened activity of subcortical structures and reduced feedback from hypoactive frontal structures. This ultimately results in exaggerated emotional responses.

The brain networks involved in ER have been extensively investigated over the last decades. One key part of ER-associated networks is the functional connectivity (FC) between the prefrontal cortex (PFC) and the limbic system (e.g., the amygdala, the hippocampus, and the ventral striatum) [45,106,107]. The automatic generation of emotions and emotional sensitivity depends on the activation of various limbic structures, which send excitatory projections to frontal regions. In turn, those regions integrate information and send feedback responses that modulate (down-regulate) emotional states [82] (see also Figure 3). One proposed simplified model of this system consists of two feedback loops that each underlie different subprocesses of ER [82]. The first one is the ventral system, which includes vmPFC, ACC, and orbitofrontal cortex (OFC), and mediates automatic ER subprocesses [82]. Meanwhile, the dorsal system involves the later-developing lateral prefrontal cortices (vlPFC and dlPFC) and mediates voluntary regulation of emotions [82] and sophisticated cognitive processes [45]. However, a finer examination of these circuits is beyond the scope of this work and results are extremely sensitive to study design and sample type. Therefore, we will not delve any further into this matter.

In Table 2, we report the main studies using functional magnetic resonance imaging (fMRI) to measure the activity of ER-associated regions while monitoring peripheral inflammatory markers [108–112]. Multiple pieces of evidence from resting state functional connectivity (rsFC) studies on adults suggest that, during peripheral inflammation, frontal cortical regions exert a reduced inhibition of the amygdala compared with non-inflammatory states [109,110]. However, other works suggest different outcomes and indicate a positive link between peripheral inflammation and regions engaged in ER during emotionally evocative tasks [108].

Findings on adolescents are also controversial. On one hand, evidence from rsFC suggests that the association between inflammatory signals and frontolimbic FC is present at younger ages [110], although other findings from emotional-eliciting tasks seem to suggest no association [112]. Furthermore, one work on 70 healthy adolescents reported that tumour necrosis factor-α (TNF-α) (but not CRP nor IL-6) was negatively associated with cortico-cortical FC between the left parietal cortex and IFG and positively associated with subcortical-subcortical FC between the amygdala and left striatum [113]. Despite the difficulty of framing these findings in a coherent picture, one possibility is that the association between inflammation and fronto-amygdalar FC undergoes directional changes across development [112], which parallel developmental modifications in neural correlates of ER and preferred regulation strategies. Significant changes in ER-associated areas are reported during adolescence [46], including a shift from positive to negative functional coupling between the PFC and the amygdala [114,115]. Interestingly, a parallel shift from the use of suppressive strategies to ones relying more on cognitive control (such as cognitive reappraisal) is also reported across development [116], which could reflect the changing neural correlates. These entail, for example, greater activation of the amygdala and other subcortical regions during ER processes in adolescents, in contrast with more substantial involvement of higher-order frontal brain regions in adults [46]. The association between inflammation and FC may therefore evolve with development. Emotional stimuli in younger subjects may trigger a prevalently subcortical response, with heightened amygdala reactivity, increased stress response, and elevated peripheral inflammation (induced through the HPA axis or other neuroendocrine pathways). In contrast, emotional stimuli in adults might elicit responses mostly driven by higher cortical structures, which downregulate amygdala reactivity, stress, and inflammation. However, as most of the studies investigating this topic are cross-sectional [109–111] and cannot ascertain causal relationships, reverse causality is also possible. In other words, brain regions may be variably sensitive to inflammation-dependent alterations based on the individual’s developmental period. Additionally, in most of the studies examined some potential confounding factors that may affect participants’ inflammatory status, such as body mass index (BMI), were not considered; these results should thus be interpreted with caution.

In general, evidence from these studies suggests a complex association between structural and functional abnormalities in ER circuits and peripheral inflammation markers, but definitive interpretations have yet to be reached.

### Peripheral and central inflammation impact brain circuitry and vice versa: potential mechanisms

As mentioned previously in this work, a vicious cycle may exist, where stress and peripheral inflammation induce neuroinflammation in the brain, disrupting cerebral connectivity and increasing, for instance, amygdala reactivity. This cycle would result in altered emotional responses, which in turn increase stress-induced local and systemic inflammation and exacerbate the impairment of brain networks via multiple routes, as summarized in Figure 2. Although the mechanistic nature of these interactions has not been fully elucidated yet (and a detailed presentation of the associated literature is beyond the scope of this review), in this subsection we discuss findings from animal and human studies that offer preliminary, mechanistic insight into how peripheral and central inflammation can affect brain circuitry.

As foreshadowed previously, the effects of peripheral inflammation on brain networks could be mediated by neuroinflammation, as the two are mechanistically linked via cellular, neuronal, and humoral routes [117,118], and inflammation has been suggested to be implicated in several psychiatric and neurological conditions [119,120]. One potential pathway involves inflammation-induced neurotoxicity through the tryptophan/kynurenine axis [121–124], which has been implicated in psychiatric [124] and neurological disorders [125]. Other mechanisms concern alterations in brain neurotransmitter metabolism; for example, abnormal serotonergic and dopaminergic signalling have been implicated in EDD [52,126,127]. Further mechanisms may involve the vagus nerve, which mediates immune responses by inducing the release of neurotransmitters and activating the HPA axis in response to peripheral inflammation [128], or BBB dysfunction [129,130], as increased permeability may facilitate the translation of inflammatory markers into the brain (see also [131]). For instance, *in vitro* studies further suggest that IgGs isolated from BD patients’ sera can effectively hydrolyse human myelin basic protein [69], implying that inflammation may impair myelination and, thus, neuronal connectivity. Another possible route involves the trafficking of inflammatory myeloid lineage cells into the brain. To elaborate, preclinical models reveal that in chronic stress conditions, immature monocytes are mobilised from the bone marrow and contribute to peripheral neuroinflammatory signalling [132,133]; these cells may then traffic into the brain following an increase in monocyte chemoattractant protein (MCP-1) secreted by activated microglia in response to peripheral proinflammatory cytokines [134]. Interestingly, translational models of stress and anxiety show that microglia activation is consistent in ER-associated regions, such as the PFC, amygdala, and hippocampus [135], which could render these regions a target of monocyte mobilisation. The latter may exacerbate neuroinflammation and disrupt the integrity of FC, aggravating anxiety and stress responses. In support of this view, one previous study in stressed mice reported that preventing monocytes from trafficking across the BBB was sufficient to suppress anxiety-like behaviour [136]. Moreover, counts of immature circulating monocytes, and no other leukocyte counts, have been positively associated with a functional disruption of the ER Network (ERN) (a brain network involved in ER which includes the IFG, middle temporal gyrus, and precentral gyrus [110]) in healthy adolescents [110].

Another potential consequence of neuroinflammation is the disruption of hippocampal neurogenesis. Previous works have reported that stress-induced inflammatory mediators produced by local microglia might impair this process [137]. This may lead to hippocampal functional alterations, thus exacerbating ED and increasing stress susceptibility [138].

A further mechanism that may connect inflammation and ED involves the gastrointestinal system. For instance, stress-induced gut dysbiosis may favour microbial translocation which could, in turn, exacerbate inflammation [139]. In the presence of pre-existing impairment or alterations of the BBB, which may be the case of BD [130], this might translate into central inflammation and neuronal damage [139]. Other routes that may be involved in this process involve the vagus nerve or signalling molecules like short-chain fatty acids (SCFAs) in the peripheral circulation (see [139] or [140]). We will elaborate more on these mechanisms in the specific context of EDD in the next sections.

Despite the limited direct molecular evidence in humans, insightful findings on the mechanisms linking inflammation and alterations in ER networks derive from subjects with a history of ELS. Several lines of evidence can be marshalled to support that ELS is associated with heightened systemic inflammation [141–146], ED [77,105,147–150], and psychiatric disorders in general [151]. ELS has a deleterious impact on the maturation of limbic brain networks by altering the development of brain structures relevant to ER, inducing heterogeneous alterations in volume [152–154] and in connectivity patterns [105,109,114,154], which might translate into higher levels of ED and vulnerability to stress and inflammation. Peripheral inflammation may also mediate the effects of ELS on ED in adult subjects [109]. This supports the existence of a ‘body-to-brain’ [109] directionality of the link between peripheral inflammation and ED in ELS: the proinflammatory milieu may ultimately affect ER-associated circuits by inducing neuroinflammation via the pathways described above in this section. However, we must note that studies analysing the relationship between proinflammatory cytokines (such as IL-6) and stress-induced neural activation of corticolimbic regions have led to mixed results [70,155]. Further research is thus warranted to clarify the interpretation of the presented findings. Another proposed conceptual framework that aligns well with several of the presented results is the ‘neuroimmune network hypothesis’, which posits that ELS may act as a catalyst that potentiates the bidirectional interplay between peripheral inflammation and neural circuits associated with emotion processing and ER [156]. This agrees with the heightened sensibility to stress and inflammatory responses in ELS-exposed subjects. Finally, other works suggest that ER strategies may be used to modulate the impact of ELS on peripheral inflammation [78,100] and perceived chronic stress [150]. It could be hypothesized that more adaptive ER strategies impact the HPA axis by reducing the stress response and peripheral inflammation. In contrast, less adaptive strategies might increase emotional reactivity [77] and susceptibility to stress (via, for example, heightened amygdala reactivity), favouring a systemic proinflammatory milieu [78]. Along these lines, transdiagnostic evidence on ADHD and BPD patients [77,157] reveals that self-reported ELS positively correlates with using maladaptive ER strategies in adulthood. If the presented associations and mechanisms were true, psychological interventions focused on acquiring effective regulation strategies might improve the health outcomes of subjects with a history of ELS that express ED, as further discussed in the last section of this work.

## Emotion regulation and (neuro)inflammation in bipolar disorder

### Emotion dysregulation: a potential trait and state marker of BD

BD is one of the most widespread mental illnesses, with an estimated global disability-adjusted life year (DALY) of approximately 8.5 million [13]. It is characterised by chronic fluctuations in mood and energy through a combination of alternating manic, hypomanic, depressive, and sometimes mixed-state episodes, as well as phases of euthymic mood [29,158,159]. Manic episodes entail a period of elevated mood or euphoria, heightened energy, and impulsive behaviour, while depressive episodes are distinguished by feelings of sadness, low energy, and apathy [160]. Two main types of BD are typically discussed: BD I (in which patients experience at least one episode of mania) and BD II (in which patients experience at least one episode of hypomania and one of major depression) [158]. Difficulties in ER on several levels are the norm rather than the exception in BD patients [3,161], and ED is a recurrent characteristic of both inter-episodic and euthymic periods [162]. It is also a predictor of greater mania symptoms [163,164]. In line with these results, some researchers have recently proposed an emotion-based model for BD pathophysiology to complement the actual, mood-based one [165]. Indeed, ED in patients with BD is further associated with cognitive and executive function alterations and poorer life quality [166,167]. Common manifestations of ED in BD patients are summarized in Table 1.

Cogent evidence supports that BD is associated with symptoms indicating ED or maladaptive ER strategies. In a recent systematic review and meta-analysis of 28 studies comparing BD patients to healthy controls (HC), patients with BD were found to utilise many more maladaptive ER strategies, including rumination, risk-taking behaviours, dampening, and negative focus in response to negative affect [161]. The effect sizes of those changes were overall smaller in euthymic patients. Interestingly, this finding seems somewhat more specific to maladaptive strategies, as the same meta-analysis reported that the utilization of most adaptive ER strategies did not differ significantly between HC and BD [161]. In another systematic review, authors found that individuals with BD and even those at risk for BD display ER impairments in both subjective behavioural measures and objective measures such as MRI [168]. An additional meta-analysis focusing on the use of the DERS questionnaire to assess ER in BD patients and HC showed that patients performed worse across categories including non-acceptance, goal-directed behaviour, impulse control, strategies, and emotional clarity [169]. Some authors also posit that the altered emotional reactivity that characterizes BD patients leads them to recur to ER strategies more frequently than HC and to perceive a reduced efficacy of these strategies, which may partly explain the higher scores at ED tests based on self-assessment (see, for instance [167]). Interestingly, there is no definitive conclusion on whether ED was a risk factor for disease development or an outcome of the disease's affective episodes [168]. Independent of the directionality of these associations, abnormal ER and heightened emotional reactivity appear to be key components of BD.

Reviews and meta-analyses have also examined several potential neural biomarkers of ED in BD [43,170–172]. Some well-documented abnormalities include altered volumes in frontolimbic networks that greatly impact the amygdala, hippocampus, PFC, ACC, and insula [173]. However, findings across studies do not always correspond on the directionality of these changes [172], and the frequent presence of lithium medication, which has been tied to volumetric brain alterations, complicates the interpretation of results [170,171,173,174]. Furthermore, diffusion tensor imaging (DTI) studies assessing white matter alterations in BD have also revealed abnormalities in white matter tracts largely involving frontolimbic structures such as the PFC and cingulate regions, among others [175–177]. Moreover, although the topic is still underexplored, brain alterations may differ between BD subtypes [175,178] and mood states [173].

Broadly, abnormalities in the above structures seem to translate into a reduced cortical control of limbic structures during ER, despite a hyperactive ventral-limbic FC [3,38,82,167,168,170–172,179], which may be responsible for the emotional lability of BD patients, as shown in Figure 3. This finding is supported by systematic reviews and meta-analyses [3,38,82,168] and is consistent with evidence from other EDD (see the dedicated sections). However, due to the substantial variation in experimental designs and definitions of regions of interest across studies, sometimes the results yield conflicting conclusions, therefore we have yet to elucidate the full picture (see, for example [179,180],). Interestingly, preliminary works also suggest that brain alterations measured in BD patients may be present in subjects at high risk of developing the disorder [181]. A genetic effect was also investigated via a study looking at healthy BD offspring who had at least one parent diagnosed with BD versus HC: relative to HC, the children who had a parent with BD presented alterations in the frontolimbic system, including reduced downregulation of the amygdala response from the vlPFC after exposure to face distracters [182]. This is coherent with another finding from a recent meta-analysis, reporting that non-affected first-degree relatives of people with BD display a greater use of maladaptive ER strategies compared with HC [161]. Alternative works point towards different brain networks also being implicated in ED: for instance, one recent study investigating ER circuitry found patterns of elevated neural activity in BD patients as compared with HC during ER task performance in the left dlPFC, left hippocampus, and right somatosensory cortex [178], consistent with an involvement of the somatomotor network (SMN) in ED, as further discussed later in this section.

While these results suggest that ED is a pivotal BD symptom with important implications for clinical care, BD neuroprogression, and potentially BD prevention and/or intervention, it is still unclear at what point ED manifests during the disease course. Whether it operates solely as an early predictor of BD or also results in part from the illness, our aim is to unravel some of the mechanisms of ER via biological factors like inflammation, with the goal of better understanding this complex relationship and its implications for the prevention and treatment of BD.

### Inflammatory markers, sleep, and gut health: the effects of inflammation on BD

While BD pathophysiology remains ill-defined, a growing body of literature points to the involvement of immune dysfunction in its mechanisms [60,183,184]. This involvement is also supported by the epidemiology of BD, as the disorder often co-occurs with inflammatory and autoimmune illnesses including psoriasis, ulcerative colitis, asthma, rheumatoid arthritis [185,186] (for a previous meta-analysis on this topic see [187]), just like other EDD. Neuroendocrine and metabolic comorbidities such as high BMI, dysglycemia and metabolic syndrome are also observed in BD patients more frequently than HC [188,189], and underscore the importance of investigating the inflammatory milieu in these patients.

A first approach to disentangle the role of inflammation in BD is to investigate its aetiology, which seems to implicate proinflammatory conditions. Some authors found that prenatal maternal infections are risk factors for BD [190,191] and affective psychosis in general [192]. Perinatal infections might also confer risk for the disorder, as reviewed in [193]. In principle, exposure to pathogens could result in hyperactivation of the immune system, potentially exerting non-specific effects on neuronal growth and survival during crucial periods for the development of brain regions and circuits. However, the quality of evidence is weak [55,193] and the effect of these infections on inflammation remains unclear (see, for example [194]). The high heritability of the disorder, estimated to be approximately 60–80% [55], has also highlighted the possibility of genetic signatures in BD. Although results about genetic markers of the disorder are still inconclusive, genetic testing of patients has identified polymorphisms associated with mediators of immune response and inflammation, including TNF-α and IL-6, which have been associated with BD and earlier disease onset [194–196]. Furthermore, the investigation of epigenetic markers has revealed that specific methylation profiles in genes for brain-derived neurotrophic factor (BDNF) are present in young subjects at high risk for BD [197]. BDNF is a well-established neurotrophic factor involved in neurogenesis that has shown anti-inflammatory properties in preclinical models [198]. Its intricate relationship with neuroinflammation may contribute to the physiopathology of various psychiatric disorders [199]. Several works indicate that BDNF may play a role in BD pathogenesis [83] and neuroplasticity changes associated with the disorder [200]. As concerns other experimental designs, promising evidence comes from Mendelian randomization studies [201], with one work suggesting a causal implication of the proinflammatory CRP and the risk of developing late-onset BD in a cohort of approximately 79,000 participants [202].

ELS, such as trauma, has also been shown to increase the risk for BD [55,203] as class II evidence [193], and lead to worse symptoms, disease progression, and more severe comorbidities [204]. Although correlation does not imply causation, since the onset of BD typically comes after any traumatic childhood experiences, one possibility is that ELS, and in particular emotional traumas, increase the risk of BD or are implicated in its pathophysiology. Detrimental effects of ELS bidirectionally connected with immune activation are presented in the previous section. Another putative mechanism investigated in BD may involve lowered lipid-associated antioxidant defences, which are tied to increased oxidative stress and inflammation [205].

In recent years, repeated efforts have been done to investigate the link between BD and both peripheral and central inflammatory mediators. Several individual research studies and previous works reported that blood concentrations of TNF-α, soluble TNF receptor 1 (sTNF-R1), and IL-6 are increased in BD patients compared with controls [206–213], as well as CRP [214–216], interleukin-1β (IL-1β) [206], soluble IL-2 receptor (sIL-2R) (related to T-lymphocyte activation) [212], interleukin-8 (IL-8) [213,217] and interleukin-18 (IL-18) [211], among others. One work worth mentioning is a study from 2019 including the most common psychiatric disorders, which summarized the result of previous different meta-analyses and reported that IL-4 and soluble IL-6 receptor (sIL-6R) are consistently elevated only in BD (but not in major depressive disorder [MDD], schizophrenia, and autism spectrum disorder). Also, sIL-2R and sTNF-R1 were found to be consistently heightened in BD, alongside IL-6 and CRP, whose elevation was measured in most disorders [218]. However, more recent meta-analyses and systematic reviews have not identified any robust pattern of peripheral inflammatory markers associated with BD [83,209]. Indeed, some of the associations discussed above are not encountered or presented in the opposite direction in other works [83,209]. One exception seems to be IL-6, whose concentration has been reported to be increased in all mood states by a recent meta-analysis [209]. This finding has led to its proposal as a potential trait marker of BD [209], and therefore deserves further investigation.

The heterogeneity of some results across systematic reviews suggests that although chronic low-grade inflammation may be a trait feature of the disorder, BD patients may undergo fluctuations in peripheral inflammatory markers also across manic, depressive, and euthymic states. Higher concentrations of IL-6, TNF-α, sTNF-R1, and CRP have been reported during mood states [194,209], with some works suggesting opposite cytokine patterns between manic and depressive phases [219]. Episodes of mania seem especially characterized by heightened proinflammatory cytokines [194,216,220]. Manic BD subjects also seem to have greater white platelet, neutrophil, and monocyte counts and higher monocyte-to-lymphocyte and neutrophil-to-lymphocyte ratios [221,222], which may indicate peripheral inflammation. Interestingly, one previous cross-sectional study found that during manic episodes, the levels of peripheral markers of oxidative damage were sometimes as high as those in patients with sepsis [223], implying that these episodes may also be characterized by severe systemic toxicity. However, a recent systematic review and meta-analysis including 51 studies on BD has not detected inflammatory markers of mood states [83]; therefore, these results should be only considered preliminary and not conclusive. Interestingly, BDNF has been proposed as a state marker of mood episodes in BD [224]. Along these lines, the previously mentioned meta-analysis examined and found a negative correlation between circulating levels of BDNF and the severity of depression in BD [83].

In addition to peripheral ones, certain neuroinflammatory mediators have also been identified in BD, although research is limited. Cross-sectional case–control studies have revealed that some BD patients exhibit elevated cerebrospinal fluid (CSF) levels of IL-1 β, IL-6 [225], MCP-1 [226] (discussed in the previous section), and YKL-40 [226], a protein expressed by neurons, astrocytes, and especially microglia, whose levels increase in chronic inflammatory conditions [227]. As discussed for peripheral mediators, central inflammatory markers might also reflect state fluctuations in BD patients. For instance, one study showed that only BD patients with a recent manic or hypomanic episode had elevated CSF levels of IL-1β [225]. Despite the promising findings, these results are only preliminary. Indeed, the diversity of experimental designs and variability of results do not allow us to infer robust associations or causal mechanisms, as also confirmed by a previous meta-analysis on postmortem studies of neuroinflammatory correlates of BD [57], which could not identify any replicable marker.

Overall, the presented findings from peripheral and central inflammation support that cytokines and inflammatory mediators may vary across disease phases and should be interpreted within a patient’s BD cycle [219]. However, evidence remains inconclusive, highlighting the need for controlled studies to identify precise peripheral biomarkers for the disorder.

Another factor of interest in BD inflammation is dysfunctional sleep. Sleep and circadian disturbances are commonly observed in BD [228] and subjects at risk for the disorder [229]. Sleep impairments may be bidirectionally connected to cortisol alterations and increased inflammation which, as mentioned in the previous sections, are part of the downstream effects of HPA axis activation. In the general population, the association between sleep impairments and peripheral inflammation is well-established [230]. BD patients demonstrate increased HPA axis activity [231], which can impact sleep cycles and quality, although the precise association with mood states is still up for debate [231–233]. In the context of dysfunctional sleep, dysregulated cortisol production, inflammation, and alterations in brain neurotransmitter metabolism may have an adverse effect on BD symptoms, as confirmed by various studies [210,234–236]. Interestingly, one recent study on 162 adults with BD reported that the dysregulation of cortical rhythm was a mediator between ELS and suicidal ideation, depressive symptoms, and insomnia [237], and another indicated that insomnia could predict several BD symptoms and ED [238]. This evidence highlights that future works should assess circadian dysregulation in the context of ELS, inflammation, and BD symptoms, including ED.

There is also mounting evidence to suggest that dysregulated or imbalanced gut microbiota can increase inflammation in the body and the brain and affect various mental illnesses, including BD [130,139,239]. One of the putative mechanisms connecting the gut, inflammation, and the brain is that gut dysbiosis induces alterations in GI permeability, allowing bacteria and proinflammatory products of their metabolism into the blood. Their presence could trigger or enhance peripheral inflammation, potentially weaken the BBB, and affect brain circuits directly through translocation or indirectly through inflammation, exacerbating a vicious cycle of heightened inflammation and consequent structural and functional damage [139,239]. In the context of BD and other neuropsychiatric disorders, this process could be exacerbated by a pre-existing hyperpermeability and dysfunction of BBB [130,139]. In support of this hypothesis, certain studies have found that patients with BD (in comparison to HC) have higher markers of bacterial translocation from the intestinal lumen [240,241]. These markers may precede and reflect an activated innate immune state [240,241]. In a previous cross-sectional study of adults with BD, a negative correlation was discovered between microbial alpha diversity and length of illness [242]. Furthermore, several bacterial clades were associated with an inflammatory state [242]. However, much is still unknown about the effect of acute BD states on gut microbiota, given that all the patients in this study were in an episode of bipolar depression. Furthermore, some studies have found higher counts of various bacteria in BD patients compared with HC, and several mechanisms have linked gut microbiota to the activity of the HPA axis in BD and other disorders [139,243]. Findings favouring an association between inflammation and GI disturbances in BD come from a previous umbrella review, which revealed that irritable bowel syndrome (IBS), which is frequently associated with heightened peripheral inflammation [244], was identified as a potential risk factor for the disorder, meeting class I criteria [193]. This association seems generalizable to mood disorders, as also observed in patients with MDD [245]. Nevertheless, there is preliminary evidence indicating higher IBS rates in BD patients with history of severe ELS, while in patients with MDD the IBS prevalence remains the same regardless of ELS history or severity [246,247].

Considering the presented evidence, examining inflammation and its associated disturbances in the context of BD pathophysiology seems crucial. Part of our aim in this review is to better understand the broad implications of inflammation on BD and the interplay between inflammation and ED as an enduring feature of the disorder, which is the focus of the next section.

### Inflammatory correlates of behavioural and neurobiological markers of ED in BD

Whether the increased inflammation which seems to characterise EDD is a cause, effect, or merely an epiphenomenon [60], this work aims to better understand the mechanisms behind this relationship and characterize its associations with ED. Several studies have investigated potential inflammatory markers and neural correlates of ER in BD [21], although the topic remains underexplored.

The evidence linking ED-associated behaviours and inflammation in BD is currently limited, but interesting results come from studies investigating emotional reactivity, presented in Table 3. One previous cross-sectional work on 613 BD patients found higher peripheral levels of hsCRP in patient subgroups characterized by abnormal emotional hyper-reactivity and hypo-reactivity (using the Multidimensional Assessment of Thymic States test) compared with normal reactivity patients [214], even after correction for potential confounding factors such as lithium medications. The authors, therefore, proposed that CRP should be investigated as a candidate marker of ED in BD [214]. Moreover, in one recent work on a cohort of 1072 adult BD patients, the same authors built a data-driven ‘allostatic load index’ including biomarkers of inflammation (CRP and albumin), cardiovascular risk (diastolic and systolic blood pressure), metabolism of lipids (triglycerides), and metabolism of glucose (fasting glucose), which could predict with 81.1% accuracy if the patients presented non-elevated or elevated emotional reactivity [103]. Notably, the subjects with predicted emotional hyper-reactivity were also the ones with poorer cognitive functioning and overall functioning, independent of other confounding covariates. These promising results suggest that the index was able to capture clinically relevant aspects of the disorder, further emphasizing the link between altered emotional reactivity (which is a starting point for altered ER processes) and integrative measures of body dysfunction, including inflammation. However, the cross-sectional measure of the study prevented the authors from assessing the directionality of these mechanisms [103].

**Table 3:** Main articles linking peripheral inflammation with emotion regulation, emotion reactivity or associated behaviours in bipolar disorder, borderline personality disorder, and attention deficit/hyperactivity disorder patients

	Diagnosis	Main Findings	Sample	Measure(s) of ED or associated behaviours	Measure(s) of inflammation	Study type	Covariates	Directionality of the findings
Dargél et al., 2017 [214]	BD	Peripheral levels of hsCRP were higher in patients with emotional hyper-reactivity and hypo-reactivity compared with patients showing normal emotional reactivity	613 remitted BD patients; mean age = 41.2, SD = 12.4	Multidimensional Assessment of Thymic States	Peripheral hsCRP	Cross-sectional	Age, sex, years of education, age at BD onset, BD subtype, total number of mood episodes (manic and depressive), suicide attempts, depressive symptoms, anxiety symptoms, manic symptoms, medications (antidepressants and benzodiazepines)	Positive correlation between hsCRP levels and alterations in emotional reactivity (hypo- or hyper-reactivity)
Dargél et al., 2020 [103]	BD	A data-driven allostatic load index including peripheral measures of inflammation (hsCRP and albumin) could predict with an 81.1% the presence of either hyper-emotional reactivity or non-hyper emotional reactivity in a cohort of BD patients	1072 BD patients with non-hyper emotional reactivity (*n* = 528) or hyper-emotional reactivity; (*n* = 544); mean age of non-hyper reactive patients = 42.05, SD = 12.67; mean age of hyper reactive patients = 41.45, SD = 12.82	Multidimensional Assessment of Thymic States	BALLI index for allostatic load including peripheral measures of inflammation (hsCRP and albumin), cardiovascular risk (systolic and diastolic blood pressure), glucose metabolism (glucose fasting), and lipid metabolism (triglycerides)	Cross-sectional	Age, sex, and smoking controlled for when assessing the allostatic load scores of patients with hyper-emotional reactivity and non-hyper emotional reactivity.	Emotional hyper-reactivity correlates with higher scores on the allostatic load; the BALLI index could predict with an accuracy of 81.1% patients with hyper-emotional reactivity
Saccaro et al., 2023* [250]	BD	Overall, structural and functional correlates of inflammation in bipolar disorder revealed a link between brain abnormalities and peripheral inflammatory markers in BD patients	Final number of studies = 23 (functional MRI = 6, structural MRI = 16, botch functional and structural MRI = 1)	MRI and functional MRI	Peripheral inflammatory mediators (depending on the study)	Systematic review	Most of the studies accounted for potential confounding factor (e.g., age, sex, treatments, and medical comorbidities)	Heterogeneous results, broadly supporting a positive link between peripheral inflammation and brain alterations in areas involved in somatomotor processing and affective processing
Westling et al., 2011 [403]	BPD	Increase in IL-1β levels in response to glucose was significantly greater in patients than in controls. Furthermore, IL-1β reactivity was associated with symptoms of hostility	13 young women with BPD and 13 healthy young women as controls matched for age and BMI range; age range = 19–24	Aggression Questionnaire Revised Swedish Version	Peripheral IL-1β, TNF-α, and IL-6	Cross-sectional	Age and BMI	Positive correlation between IL-1β levels and glucose intake during a 5h glucose challenge (higher in BPD patients than controls). Additional positive correlation between IL-1β reactivity and symptoms of hostility
Yang et al., 2020 [329]	ADHD	Higher peripheral CRP levels were associated with more severe ED, including the total ER scale and the strategies subscale	105 adult ADHD patients and 57 healthy adults as controls; median age of ADHD patients = 36 (age range = 29–43), median age of controls = 38 (age range = 34–43)	DERS-16	Peripheral CRP, SAA, sICAM-1 and sVCAM-1	Cross-sectional	Age, sex, BMI, ADHD medication, and other medications	Positive correlation between ED and CRP levels, no correlation between ED and SAA, sICAM-1 and sVCAM-1 levels
Skott et al., 2020 [356]	ADHD	The administration of a synbiotic for nine weeks improved ER in the subgroup of adults with high baseline sVCAM-1 levels	114 adult ADHD patients, 57 receiving the synbiotic and 57 receiving the placebo; median age = 36 (age range = 29–42)	DERS-16	Peripheral SAA, sICAM-1, and sVCAM-1	Experimental	Age and sex	No effect of the synbiotic on DERS score after stratification for medication status. Positive effect of the synbiotic on DERS score in the subgroup of patients with high sVCAM-1 baseline levels

Studies marked with ‘*’ are reviews. Abbreviations: ADHD, attention deficit/hyperactivity disorder; BALLI, allostatic load index for BD; BD, bipolar disorder; BMI, body mass index; BPD, borderline personality disorder; CRP, C-reactive protein; DERS-16, difficulty in emotion regulation scale-16; ED, emotion dysregulation; ER, emotion regulation; hsCRP, high-sensitivity C-reactive protein; IL-1β/-6, interleukin-1β/6; SAA, serum amyloid A; SD, standard deviation; sICAM-1, soluble intercellular adhesion molecule 1; sVCAM-1, soluble vascular adhesion molecule 1; TNF-α, tumor necrosis factor-α.

Meanwhile, other individual studies have sought immune activation markers in ER-associated regions with promising results. Authors have found increased activity of 18-kDa translocator protein (TSPO), a marker of immune response and microglia activation, in the hippocampus of euthymic BD patients using positron emission tomography (PET) imaging [248]. In another study by the same group, the hippocampal concentrations of N-acetyl-aspartate (NAA) and N-acetyl-aspartyl-glutamate (NAG), markers of neuronal integrity related to microglial activation [57,72], were increased in BD compared with HC [72]. Moving to another direction, one recent work points to the involvement of ceramides in BD neuroinflammatory processes [249]. This previous review reported that BD patients displayed high levels of ultralong-chain ceramides and galactosylsphingosine in the cortex, substantia nigra, and nucleus accumbens, as well as elevated serum levels of sphingolipids [249]. One tentative hypothesis posits that ceramide enhances the FC between the nucleus accumbens core and PFC, favouring defective myelination and enhancing neuroinflammation.

Some interesting lines of evidence come from studies assessing MRI correlates of peripheral inflammatory markers simultaneously in BD patients. Although preliminary, the literature suggests a qualitative trend toward increased proinflammatory marker concentrations and impairments in ER-associated networks, as summarized by a recent systematic review including 23 studies from MRI and fMRI correlates of peripheral inflammation [250]. Notwithstanding the acknowledged limitations arising from a restricted number of studies and smaller sample sizes, the work sheds light on qualitative trends that could pave the way for further research endeavours. As concerns structural MRI, several works supported a negative correlation between peripheral inflammatory mediators and the volume of brain areas involved in emotion processing and regulation [251–254]. According to the same systematic review [250], results from fMRI studies follow a similar trend. For instance, FC-based investigations suggest negative [255] or no correlation [213] between peripheral levels of IL-6 and rsFC in areas implicated in ER. In a previous investigation, it was found that adult BD patients’ levels of circulating cytokine-producing NK cells were higher compared with HC [256]. These levels were also tied to heterogeneous alterations of the activity of brain regions linked to emotion processing and regulation, such as the dlPFC, hippocampus, cuneus and precuneus, as well as the amygdala-temporal pole FC and the amygdala-parahippocampal gyrus FC [256]. To explain these results, the authors hypothesized an intricate pathway linking lymphocyte and peripheral cytokine fluctuations with brain demyelination, although lithium medication may have also played a role in these findings [256]. As also highlighted by the authors, some of these brain regions have previously been connected to specific indicators of ED, including suppression of negative emotions, rumination, and suicidality [257–261]. Of note, suicidality has also been linked to specific neuroinflammatory markers in BD I; even after controlling for working memory and inhibitory control, serum levels of sTNF-αR1 were significantly higher in BD patients with suicidal ideation than those without [262]. Similarly, there is a relationship between the severity of psoriasis, a chronic inflammatory condition, and the risk of suicide attempts [263].

Alongside brain areas primarily involved in ER-associated circuits, the inflammatory state may also affect the SMN, associated with somatomotor processing. The disruption of the SMN has been linked to emotion processing difficulties in BD patients [213], although the exact mechanisms behind this relationship are not yet fully understood [213,264–267]. One hypothesis is that sensory processing, or how a sensory stimulus is perceived and assigned importance or saliency, may underlie ED as a component of the ER process [268]. A decrease in SMN-associated FC is documented in depressed BD patients [269,270], and inflammation may also play a role during depressive episodes. Indeed, proinflammatory interleukins (IL-6, 8) have been associated with hypoactive SMN in depressed BD patients (on both pre- and postcentral gyri) [213,255]. One tempting speculation based on these results is that depressive states, characterized by apathy and reduced psychomotor activity, could be associated with reduced activation of somatomotor areas [250], although the precise link between inflammation and ER is still unclear.

Finally, preliminary insights are also offered by transcriptomics studies which have been conducted to explore the molecular basis of BD [271–275]. These works have revealed alterations in gene expression patterns in specific brain regions associated with mood regulation, such as the prefrontal cortex, anterior cingulate cortex, and hippocampus [276–280]. The genes found to be differentially expressed have offered valuable clues about dysregulated pathways [271,280], including those related to synaptic plasticity, neuroinflammation, and circadian rhythms [281–283], and point to the importance of researching both coding and non-coding RNAs [275], although results remain inconclusive. Direct evidence has been provided by recent work investigating the expression of immune-related genes in over 2000 post-mortem brains from controls and patients affected by six neurological or psychiatric conditions, including BD [284]. According to this study, most of the patients presented altered brain expression of immune-related genes compared with controls. The immune-related changes were primarily associated with innate immune activation as well as stress response, including a down-regulated expression of the genes for the corticotropin-releasing hormone, which controls the peripheral production of cortisol and can act as CNS neuromodulator in inflammatory states, and Tachykinin Precursor 1, tied to altered immune responses of astrocytes and microglia [284].

To recapitulate, while much remains to be investigated about the link between inflammation and ED in BD, there are preliminary indicators of an association between specific markers and behavioural and neuroanatomical changes in regions associated with ER. The presence of confounding factors such as lithium medications, which may impact both inflammation and neural correlates of ER, further complicates the picture [256]. Nevertheless, manic or hypomanic episodes might be beneficial moments to examine correlates of ED, given the high sensitivity to inflammation during these periods demonstrated by multiple studies. In future works, BD patients should not only be compared by their mood stages but also by specific symptoms, such as rumination and suicidality, which can have a significant impact on neuroinflammatory markers.

## Emotion regulation and (neuro)inflammation in attention deficit/hyperactivity disorder

### Emotion dysregulation is a core feature of ADHD with crucial clinical implications

ADHD is a highly prevalent neurodevelopmental disorder, with an estimated DALY of 1.03 million [13]. It manifests through features of inattention, hyperactivity, and impulsivity, which frequently persist into adulthood [28,285,286], affecting approximately 5–7% of youth [287] and 2–3% of adults [288]. However, data on prevalence vary significantly across studies [286,287,289]. While accounting for this dramatic heterogeneity, ED has been consistently associated with this neurodevelopmental disorder at all ages [290–301] and is currently a topic of extensive scientific investigation. In the context of ADHD, the construct of ED often expresses through (but is not limited to) irritability [302], i.e., proneness to anger. Other expressions of ED in the context of this disorder are summarized in Table 1. Moreover, ED mediates the association between ADHD and a wide range of symptoms in youths [290,303] and adults [304], including overall functional impairment, social impairments, and depressive symptoms. Interestingly, recent genome-wide association studies (GWAS) demonstrated that ED should not be considered a mere comorbidity of ADHD, being associated *per se* with genetic liability for the disorder [305]. In view of these and similar findings, it has been proposed that ED is a core aspect of ADHD [296,306,307] and that ADHD should be included among the EDD [5]. Some authors also suggest that ED should be seen as the fourth dimension of ADHD and integrated into its diagnostic criteria [297,308,309].

The use of maladaptive ER strategies in ADHD subjects compared with HC is well-documented. As also supported by a recent systematic review [296], ADHD patients seem to make less use of cognitive reappraisal and rely more on expressive suppression (see also [298,299,301,310]), which is not ideal given that cognitive reappraisal has been frequently tied to positive health outcomes while expressive suppression has been often associated with adverse effects on health (see earlier sections). However, the trends in these two ER strategies in ADHD patients are still debated (see, for example [301]). One hypothesis is that ADHD patients discard cognitive reappraisal because it proves less effective in these subjects due to underlying executive functioning deficits [301]. Expressive suppression may be thus used as a compensatory strategy, owing to its beneficial effects in the short term [301]. Individuals affected by ADHD also seem to rely extensively on situational and cognitive-behavioural avoidance [298,304]. Importantly, in the context of ADHD, maladaptive ER strategies have been tied to more severe symptoms, as indicated by a previous meta-analysis [297].

On a neuronal level, multiple alterations in ER-associated circuits have been documented in ADHD patients. Interesting results come from transcriptomic studies. Works on ADHD patients have primarily focused on identifying gene expression changes in the prefrontal cortex, striatum, and other brain regions involved in attention and impulse control [311–313]. These studies have provided evidence of altered expression levels of genes related to neurotransmitter signalling, synaptic function, and neuronal development [314] but have not been able to identify unique signatures of the disorder.

Neuroimaging evidence reports structural or functional abnormalities in the PFC and frontostriatal pathways [44,301,315–317], the amygdala [301,315,318,319], and the insula [318,320,321], among others, although evidence is highly heterogeneous, and systematic reviews or meta-analysis often point towards mixed results [44,300]. This variability possibly depends on multiple factors acting on separate levels, including the heterogeneous ADHD presentation, experimental settings (i.e., resting-state versus task-based), age of participants, comorbidities, medication state, and evaluation strategy for ED. Moreover, the reduced sample sizes often limit the statistical power of the analyses [44]. Broadly, abnormalities in these brain regions seem tied to ED transdiagnostically since heightened emotional reactivity and reduced top-down control of emotions have also been reported in other EDD, as discussed in other sections of this work. Some intriguing results have emerged from a recent investigation on the neural correlates of irritability, which, as foreshadowed above, is strongly associated with emotional lability and ED in these patients. One previous work reported that the rsFC between the amygdala and the IFG was altered in 34 ADHD adolescents and young adults (age range = 12–23) compared with 34 controls, with down-regulated inhibition of the amygdala from the IFG in the former group [319], supporting reduced cognitive control of emotional responses and altered reward processing, which may also play a role in irritability.

Recent longitudinal work on 104 ADHD adolescents and young adults (age range = 12–27) has moved one step beyond correlational analysis and proven a possible directionality in the interplay between ADHD and ED, suggesting that ED may predict changes in the severity of the disorder [320]. The study measured the efficiency of ER-related brain networks at two different time points and reported that individuals with higher levels of ED in adolescence experienced more severe ADHD symptoms in adulthood, independent of the baseline severity for the disorder. In contrast, improvements in ER were associated with a better course of the disorder, even after corrections for possible confounding factors [320]. The evidence that dysregulated emotional networks confer risk for increased ADHD severity is also coherent with combined prevalence data. Indeed, although the prevalence of ADHD is usually higher in youths [289], the proportion of ADHD individuals with high ED increases from childhood (25–45%) to young adulthood (30–70%) [322]. One may therefore speculate that ED contributes to the ADHD progression directly or via a third unobserved factor, suggesting that interventions focusing on ER strategies may ultimately play a role in modulating the disease trajectories, as discussed in the last section.

### Clinical and genetic evidence links ADHD with inflammation and immune alterations

The frequent co-occurrence of ADHD and autoimmune and inflammatory comorbidities, including eczema [323], atopic dermatitis [324], allergic rhinitis, asthma [324], and psoriasis [325] (see also [323] for a meta-analysis) has raised the possibility of a neuropathological role of the immune system and inflammation in ADHD [61,79], as in other EDD.

Results on genetic polymorphisms reveal a link between a heightened risk for ADHD and an altered inflammatory response. This connection is particularly noteworthy due to the high heritability of this disorder, estimated to be approximately 70–80% [326]. The transmission of several polymorphisms associated with inflammatory mediators has been documented, including IL-1 receptor (IL-1R), TNF-α, IL-6, and IL-2 [80], alongside genes involved in cell adhesion [327], although none of these mediators can be considered a genetic marker to date, due to the lack of robust evidence.

Other works have investigated the concentration of peripheral proinflammatory mediators in ADHD. Children affected by the disorder display higher proinflammatory cytokine levels according to previous cross-sectional studies [328] and reviews [79], whereas results from adults are contradictory but overall support a positive correlation between peripheral inflammation and ED [60,61,79,329]. Several mechanisms have been proposed to link ADHD and inflammation. One of these involves the HPA axis, like in other EDD (see the other disorder-specific sections). Indeed, several kinds of functional impairments of the HPA axis and cortisol-associated responses have been demonstrated in ADHD children [330] and adults [331], although results are sometimes conflicting, as summarized by a previous review [60]. Another potential mechanism involves complex relationships between the balance of pro- and anti-inflammatory cytokines, the kynurenine pathway, and ADHD symptoms (see [332] for further details).

One crucial context where inflammation may influence ADHD pathogenesis is early neurodevelopment. Some well-documented factors that heighten the risk of developing ADHD are maternal smoking status [333], obesity [334], and stress [335], which could induce a chronic proinflammatory milieu. Also, maternal autoimmune or inflammatory diseases seem to increase the likelihood of ADHD onset in offspring [336,337], and the risk of psychiatric disorders in general [337]. Recent work has also proven a positive association between the peripheral concentration of the proinflammatory transcription factor nuclear factor kappa B (NFκB) in 62 pregnant women in their third trimester and the occurrence of ADHD in their children when they were 4–6 years old [338]. Some preclinical works suggest an active involvement of mast cells [339] and microglia [340,341] in the local brain inflammatory processes (see also [61]), which could result from peripheral inflammation (see previous section). However, it is crucial to note that these findings are preliminary, and further replication studies with larger sample sizes are necessary to validate these results.

Childhood trauma and other forms of ELS might also be implicated in ADHD [342]. Albeit scant, evidence is accumulating that childhood emotional and physical maltreatment positively correlates with the likelihood of ADHD onset [343,344] and with the severity [345,346] or persistence [343] of symptoms, including ED [77]. The results seem generalizable to other psychiatric illnesses and EDD rather than only ADHD [77]. However, more research is needed to assess the potential mediating effect of inflammation in this association.

Overall, further investigation is warranted to better characterize the association between ADHD and inflammation and ascertain the underlying causal relationships, despite the promising results reviewed here.

### Inflammation, emotion dysregulation, and gut-brain axis abnormalities in ADHD

In the previous sections, ED and inflammation emerged as recurrent aspects of ADHD pathophysiology, with potential for diagnostic and therapeutic applications. However, limited works have searched for mechanisms or associations between these two in ADHD. In our quest to unravel the intricacies of this relationship in EDD, we now present relevant studies on the association between inflammation and ER in ADHD patients (see also Table 3).

Sparse and preliminary evidence has sought to assess the link between ED and inflammation in ADHD, which might involve the gut–brain axis. Like other neuropsychiatric illnesses, ADHD has high comorbidity with gastrointestinal disturbances, such as constipation and abdominal pain [347,348], and diseases, such as ulcerative colitis [349]. Some works have also reported discrepancies between ADHD and HC in the microbiome variability [350–352], which could be associated with distinct proinflammatory profiles and ADHD symptoms [353,354], although results are inconclusive [355].

A recent cross-sectional study investigated the bacterial strains in the gut microbiome of 54 children affected by ADHD compared with 22 HC and found that the concentrations of the bacterial strains were positively associated with emotional-behavioural symptoms which could result from (or express) ED [350]. Ruminococcus gnavus group was positively correlated with externalizing symptoms (i.e., rule-breaking behaviour) and Agathobacter with internalizing symptoms (i.e., depressive symptoms and withdrawal). Authors speculate that one mechanism behind this association may involve increased circulating levels of SCFAs, which are produced by the metabolism of some strains of these bacteria and may have neuroactive properties [354]. The complex links between inflammation, ER, and gut–brain axes in ADHD have also been explored by one previous cross-sectional study. This study reported a positive association between GI symptoms and peripheral proinflammatory cytokines, as well as between ED and blood levels of inflammatory markers in a cohort of 105 ADHD and 57 neurotypical adults [329]. Indeed, using general linear models, the authors found a positive correlation between CRP blood concentration and ED measured with the DERS score (adjusted R squared = 0.25), which persisted even after correction for age and BMI. Investigating specific ER subscales, as in the framework proposed by Gross [87] (including goals, non-acceptance, impulse, strategies, and clarity), a stronger correlation was identified between CRP and ED both on the total scale and the subscale measuring the access to effective ER strategies [329]. Of note, a positive association between CRP peripheral levels and the severity of GI symptoms was also found in the study, but this association was not analysed jointly with ED to rule out spurious correlations. Therefore, these results require careful interpretation.

On another note, a recent placebo-control study on 114 adults with ADHD [356] revealed that the use of a commercial synbiotic (i.e., a combination of prebiotics and probiotics, which are thought to have beneficial effects on gut dysbiosis) was tied to an improvement in ER in the subgroup of patients with elevated soluble vascular cell adhesion molecule-1 (sVCAM-1), which facilitates the leukocyte adhesion to the endothelium during inflammatory processes. In this subgroup, the patient treated with the synbiotic reported an improvement in four subdomains of ER (i.e., clarity, goals, strategy, and nonacceptance) compared with the placebo group while controlling for relevant covariates and nutrients. One of the possible explanations is that the effect of the symbiotic on the DERS score was somehow mediated by inflammation (via, for example, the mechanisms described in previous sections), especially in subjects with an elevated baseline level of inflammatory markers. However, the concentrations of inflammatory mediators were measured only at baseline. Thus, the causal involvement of inflammation cannot be ascertained. Moreover, some of the patients were under different medications at the time of the study, and the authors could not exclude the effect of treatment on results due to the reduced sample size of the subgroup. Notably, a previously mentioned work [329] did not find any correlation between the DERS score and peripheral level of the inflammatory markers serum amyloid A (SAA), soluble intercellular adhesion molecule-1 (sICAM-1), and sVCAM-1 after correction for relevant covariates. Further research designs should therefore rely on greater sample sizes and stratify patients by medication status and other potential confounding factors.

These results broadly suggest an association between ED and gut barrier dysfunction associated with (or induced by) inflammation in ADHD. As anticipated in the previous section, impairment in the gastrointestinal barrier could be linked to BBB damage and brain alterations via a plethora of mechanisms, including peripheral inflammation [139]. Interestingly, elevated levels of cell-adhesion molecules, documented by some of the studies presented here [329,356], may reflect vascular dysfunctions and BBB hyperpermeability [357] or impairments in BBB [358]. The hypothesis of a disrupted BBB in ADHD is also supported by a recent study, where higher levels of peripheral claudine 5, another cell-adhesion molecule present in epithelial tight junctions and a marker of BBB damage, were found in 80 ADHD children compared with 40 HC [359]. Further research and meta-analysis are ultimately needed to draw statistically sound conclusions and eventually better elucidate the mechanistic associations behind these findings, which should be seen as starting points for future investigations.

## Emotion regulation and (neuro)inflammation in borderline personality disorder

### A core feature of and contributor to BPD: emotion dysregulation

BPD is a severe mental health disorder that is present in 1–3% of the general population and is clinically the most diagnosed personality disorder [360]. BPD is characterized by instability in domains ranging from relationships to emotions and has a suicide rate of approximately 10% [6,361]. One of the core characteristics and aetiological bases of BPD is ED, which is allegedly incited by impaired social communication in infancy and worsened over time via environmental, physiological, and cognitive processes [56]. BPD patients also show alexithymia, or an inability to identify and describe emotions [362]. As in other EDD, traits of ED are believed to emerge when patients experience adversity, like psychosocial stress [363].

One way to understand these components is to view them as a dynamic and interconnected process. Linehan's biosocial theory states that emotional sensitivity is inborn for BPD patients [7]. This sensitivity potentiates negative affect, which then hinders the process of learning effective ER strategies. For instance, one of BPD's most devastating characteristics is rage, which is empirically linked to perceived rejection [364]. The Biosocial Developmental Model of BPD extends Linehan’s theory and suggests that conceptualising this disorder's development over the course of a lifetime yields a more nuanced model [365]. In patients who experience trauma, especially during childhood, affective instability may be intensified. Overall, a lack of adaptive strategies as well as maladaptive strategies result in increased emotion sensitivity and recursively reinforce ED in BPD. Finally, aspects such as rumination, metacognition, and experiential avoidance are present in a higher degree in BPD and exacerbate the long-term harm on patients [25,366]. Common aspects and manifestations of ED in BPD are reported in Table 1.

Evidence shows that childhood maltreatment is positively associated with impulsivity and ED, and that targeting ED in therapy could help decrease impulsive behaviours in BPD patients [367]. ED is also cited as a mediator of the link between childhood maltreatment (and emotional abuse) and aspects of the disorder, including symptom count and feature severity [368–370].

As concerns the neurobiological correlates of BPD, previous studies have reported abnormalities in regions associated with emotion processing and regulation, similarly to other EDD. These include hyper-reactivity of the amygdala and reduced activation of the bilateral dlPFC, which are also found in subjects displaying non-suicidal self-injury [371,372]. Interestingly, one previous review reported that dialectical behavior therapy, a widespread type of psychotherapy for BPD patients with a strong focus on improving ER strategies, reduces amygdala activation and impacts the FC and grey matter volumes of IFG, ACC, and insula [371]. These promising results suggest that the beneficial effects of psychotherapy may indeed be associated with the restoration of ER circuits.

Still, much is unknown about the neurobiological underpinnings of BPD, the unique dimensions of ED in BPD, and how different types of ED (such as interpersonal and intrapersonal) manifest [373]. Also, more studies are needed to explore how ER strategies can eventually inform treatments [373]. Thus, variables such as inflammation, which appears closely associated with BPD pathophysiology, should be considered. We will investigate these associations in the following sections.

### Inflammation in BPD: genetic and epigenetic markers, pathways, and conditions

BPD is believed to have a complex, multifactorial basis, with factors ranging from genetics and neurotransmitter levels to brain development and environmental influences. While previous work has reported a certain degree of heritability of the disorder [374], no genetic variants with genome-wide significance have been illuminated yet, and it is likely that future studies will identify risk factors amidst the genetic heterogeneity of BPD [374,375]. An altered HPA axis has also been linked with inflammation, as discussed previously in this review, as well as with several conditions, including BPD. BPD patients with a history of chronic abuse showed hyperresponsive HPA axes with enhanced cortisol responses [376]. Like BD patients, when salivary cortisol was measured as a non-invasive hormonal biomarker of HPA axis activity, BPD patients had higher basal cortisol levels compared with controls but showed blunted cortisol following psychosocial challenges [377–379]. More recently, as studies have begun investigating the relationship between inflammation and BPD, a review paper has established a bidirectional relationship between anxiety and inflammation in the disorder [21]. Inflammation has been shown to impact several regions closely associated with both anxiety and ED, including the amygdala, anterior cingulate cortex, and insula [117,318].

We have gathered direct and indirect evidence from several studies about inflammation in BPD. Multiple groups have demonstrated associations among salivary hypocortisolism, inflammatory cytokines, and stress-related chronic pain [380,381]. Pro- and anti-inflammatory cellular pathways have been identified as biological markers for BPD and other impulsive syndromes, with BPD patients showing increased cytokine expression [63]. In a study assessing a randomly selected sample of over 1,600 adults (ages 55–65) from the community, associations emerged between IL-6 and CRP, and higher BPD symptomatology [382]. Important to note is their use of a dimensional rather than categorical approach to assess BPD pathology. Both markers were linked to worse physical health, and IL-6 was also connected to childhood abuse. In BPD animal models, cytokine and stress-hormone levels are higher compared with controls [383]. These studies highlight the importance of investigating biomarkers of inflammation, like cytokines, in the context of BPD symptoms such as stress and ED.

One study investigating inflammatory and antioxidant pathway dysfunction in BPD discovered that there are decreased levels of antioxidant enzymes such as catalase, glutathione peroxidase, superoxide dismutase, and nuclear factor of kappa light polypeptide gene enhancer in B-cells inhibitor alpha (IκBα) in patients with BPD [384]. Meanwhile, levels of several inflammatory factors in those patients are elevated: NFκB, inducible nitric oxide synthase, prostaglandin-endoperoxide synthase 2, kelch-like ECH-associated protein 1, and NAD(P)H Quinone Dehydrogenase 1 [384]. Heightened levels of MCP-1 and stromal cell-derived factor 1, a chemokine protein known to regulate inflammatory responses [385], were also found both in women and in men versus HC for all types of personality disorders [386]. The levels of chemokine ligand 5, which is associated with chronic inflammation [387], were increased in men compared with the control group, and higher in women than in men. There were also higher levels of chemokines in women with BPD than men, indicating gender disparities and a need for further investigating proinflammatory interleukins as biomarkers of personality disorders.

Recent evidence shows that the concentration of 8-hydroxy-2'-deoxyguanosine, the oxidised form of guanine and a biomarker of oxidative stress burden, was correlated with the number of general symptoms of BPD in patients [388]. In the first study conducted to examine the role of protein kinase C (PKC) and BDNF in BPD, researchers found that medication-free male BPD patients had lower platelet BDNF and PKC-α levels than healthy male controls, and across both males and females, phosphorylated-PKC-α (p-PKCα) and PKC-α activity was lower [389]. Interestingly, PKC is an important mediator in inflammation and BDNF has neuroprotective and anti-inflammatory effects. These findings suggest that PKC and BDNF, which help regulate inflammation, have altered activity in BPD, just like they do in other neuropsychiatric disorders, including BD [390–392] and depression [390].

Epigenetic alterations have also been proposed as potential mediators of disease development in BPD. One study found that increased methylation at certain promoters (and thus downregulation of BDNF) is associated with stressful life experiences that affect adult psychopathology [393]. Compared with controls, BPD patients had higher methylation, which was also associated with increased childhood trauma. Patients underwent 4 weeks of intense dialectical behavior therapy, and responders to therapy had a subsequent decrease in methylation. In addition to highlighting the link between inflammation and characteristics of BPD like ER, this study showed that epigenetic marks can ultimately be altered through psychotherapy.

Preliminary results link stress-related psychological factors and personality disorders to the development of various inflammatory conditions, such as rheumatoid arthritis, a chronic inflammatory disease [362]. In a specific case, one 20-year-old female with BPD developed a case of autoimmune encephalitis, which was cited as a potential result of BPD-induced inflammation [394]. In another study, an association between hidradenitis suppurativa, an inflammatory skin disease, and alexithymia, which is common in BPD patients, was established using the Toronto Alexithymia Scale (TAS)-20 questionnaire [395]. These studies reinforce the idea of inflammation and psychological dysfunction bidirectionally impacting one another, and connect inflammation to specific features like alexithymia, which largely involves maladaptive styles of ER [396].

Evidence from studies of relevant comorbidities also supports the association between BPD and inflammation. In a study of chemokines, chemokine receptors, and IL-6 in 460 hospitalised patients (25–48 years old) with personality and panic disorders, inflammatory markers were elevated in study groups compared with controls [397]. Chemokines and chemokine receptors may ultimately be used as inflammatory markers in patients with panic disorders that coexist with personality disorders to determine disease severity. Interestingly, panic disorders were indicators of consistently maintained inflammatory activity in the immune system of patients with personality disorders [397].

### Linking inflammation and ED via neuroimaging and behaviour in BPD

As previously mentioned, neuroimaging studies have indicated that ER networks like the frontolimbic inhibitory network (described in the first section) are impaired in BPD, in addition to patients showing reduced brain glucose metabolism in the brainstem, insula, and frontal white matter [40,398]. This research suggests that metabolism decreases in these regions have larger effects on networks and ultimately symptomatology [399]. Considering the significance of ED in BPD aetiology, the link between inflammation and ED must also be investigated as a driver of symptoms in this disorder and other similar psychiatric disorders.

In the context of transcriptomic studies, evidence on BPD patients is extremely limited. One study explored blood gene expression in 31 females with BPD and found that IL-6 was positively associated with a score measuring the symptom of dissociation [400]. Of note, as indicated by prior studies, dissociation has been tied to self-injury and depression, and is more prevalent in subjects with a history of ELS [401]. However, future studies will need to rely on greater sample sizes to identify transcriptomic markers or correlates of ED and inflammation in BPD.

As far as we are aware, only two studies directly investigate the link between components of ED in BPD and inflammation. Certain BPD symptoms, such as aggression, have been linked to alterations of the monoamine oxidase-A-, the oxytocinergic-, and the prefrontal-limbic-system, as well as increases of the thyroid hormone T3, γ-aminobutyric acid and multiple other peripheral and central inflammatory markers [402]. For instance, one study found an increase in levels of IL-1β in BPD patients with aggressive behaviour compared with controls; IL-1β reactivity was also associated with symptoms of hostility [403]. These studies emphasize the link between inflammation and emotional control, as ED has been identified as an important mediator of aggressiveness, especially in BPD [404].

Preliminary evidence from animal models suggests that glial cells may be involved in regulating behaviours like self-injurious behaviours, which are a frequently seen clinical sign of BPD [405]. In one study examining self-injurious macaques, results showed increased vimentin expression on astrocytes and activation of pathways involved in neuroinflammation, tissue remodelling, and cyclic adenosine monophosphate (cAMP) signalling, indicating that glial cells are potential therapeutic targets [406]. This is significant, because findings have previously shown that the factor of ED is associated with a history of non-suicidal self-injury in BPD patients; however, this relationship is noted to be complex and precise mechanistical insight is still lacking [407].

Additional indirect clinical evidence comes from the finding that alexithymia, a symptom of BPD that is largely linked to ED [408], was more common among patients with severe atopic dermatitis (AD), which is an inflammatory disease, compared with patients with a milder form of AD [409]. Finally, suicidal risk, which is predicted by limited access to ER strategies [410], has been shown to increase because of certain skin disorder medications, such as interferons, that can lead to inflammatory responses [411,412].

Like other EDD, a prenatal aetiology for BPD has also been proposed, given the effects of prenatal stress and maternal dysbiosis, or gut microbiome imbalance, on the infant’s gut microbiome [413]. Stress dysregulation, which impacts emotional responses, is closely associated with gut dysbiosis and increased GI permeability, as well as heightened oxidative stress levels and immune-inflammatory activity [413]. A potential dysbiosis may also exist among bacteria that produce short-chain fatty acids in BPD [414]. Gut dysbiosis during development is further believed to impact the development of the amygdala and its connections with other brain regions, which is understandable given that BPD patients have a smaller right hippocampus and amygdala compared with HC [415]. These areas are involved in the ER process as well, ultimately suggesting that the bidirectional link between inflammation and ED underpins BPD in similar ways to other EDD.

## Conclusions on inflammation and EDD: similarities, differences, caveats, and perspectives

This narrative review highlights the associations between ED and peripheral or central inflammation in BD, ADHD, and BPD, three prevalent and severe EDD, which still lack physiopathological insights and effective therapeutic solutions. The reported experimental and clinical evidence mainly supports a positive link between ED and inflammation in EDD. However, although the topic is gaining increasing scientific attention, the literature is still limited, and the quality of most evidence is weak. Further work is needed to unravel the intricacies of these associations, especially in BPD. Therefore, the hypotheses presented in this review remain speculative, and additional research is warranted to answer most of the open questions.

Overall, the evidence supports that inflammation and ED are positively bidirectionally connected. On the one hand, inflammation may impact brain structures controlling ER, such as the hippocampus, amygdala, and ventral and dorsal prefrontal cortices. Mounting evidence supports that peripheral inflammatory cytokines may cause neuroinflammation and trigger stress responses through humoral, neuronal, and cellular mechanisms [117,118]. Therefore, peripheral inflammation may act indirectly on ER networks via distinct simultaneous pathways (see the second section of this work). It is also possible, however, that inappropriate ER processes and heightened vulnerability to stress result in altered brain responses to emotional stimuli. In turn, this may trigger a plethora of abnormal neuroendocrine downstream effects involving, among others, the HPA axis and sympathetic nervous system, ultimately resulting in increased central and peripheral inflammation [49] (see the dedicated section). An alternative scenario that we have not explored in this study is that peripheral inflammation and neuroinflammation might occur in parallel, representing two alternative responses from different physiological subsystems to insults or stressors. Although the insurgence of neuroinflammation by induction of peripheral inflammation has been proven by preclinical works (see, for example [416], or the recent narrative review [417]) and may occur through the mechanisms described in this review, evidence mostly comes from animal models, as it is extremely challenging to demonstrate causality in humans. Considering this information, the possibility that CNS and peripheral systems activate parallel responses to perceived threats remains viable and warrants additional investigations.

These general considerations gain considerable meaning in the specific context of EDD, as both ED and inflammation have been identified as shared features of BD, BPD and ADHD pathologies, albeit to a different extent. The imbalances in the literature (with more studies focusing on BD, a limited number of papers on ADHD, and mostly indirect evidence in BPD) and the lack of a unified framework ultimately compelled us to perform a narrative review rather than a systematic one. Despite these caveats, analysing a growing body of literature has yielded numerous promising results.

Regarding ER, several transdiagnostic correlates of behavioural and neuronal markers of ED have been identified. Subjects affected by EDD seem to rely on maladaptive regulation strategies more often than HC. Also, the degree of inefficacy in emotional responses frequently correlates with the severity of symptoms and disease outcomes. On a neural level, anatomofunctional alterations have been identified in the main areas involved in ER in EDD. Although the variability of results within and among EDD limits the possibility of inferring robust conclusions, one consistent pattern involves a reduced cortical control of ventral limbic areas, with hyperactive subcortical activity resulting in heightened sensitivity to emotional stressors and a reduced ability to control emotional responses (see Figure 3). Recent studies comparing BPD and ADHD [418,419] or BPD and BD [169] emphasize the challenge of distinguishing EDD based solely on ED since their emotional dynamics largely overlap (see also Table 1). On the other hand, some disorder-specific aspects have been pinpointed by a modest number of studies: for instance, ED seems to be more intense in ADHD compared with BD [27], and subjects with BPD seem to be characterized by higher impulsivity and more limited access to ER strategies compared with BD patients [169]. Our investigation revealed a lack of comparative studies with all three EDD, which are needed. Future research designs should also consider crucial factors that have been neglected by past experimental studies and may severely affect the interpretation of results, such as the context in which ER strategies are deployed, their outcome, and the patient’s medication status.

A proinflammatory milieu has been consistently documented in EDD, as suggested by the frequent comorbidity with autoimmune and inflammatory diseases. Inflammation has also been associated with ED, although the directionality of this association and its underlying causal relations remain largely unknown. One recurrent aspect of EDD is a history of ELS [204,420–422], which may affect ER circuits and heighten sensitivity to stress and inflammatory responses in adults, following mechanisms discussed in the second section of this review. These preliminary results can be interpreted as a positive correlation between peripheral inflammation and ED. The putative mechanisms behind these associations include, among others, gut dysbiosis and bacterial translocation [139], leading to a peripheral increase of proinflammatory cytokines, which can ultimately cross the BBB and induce neuroinflammation, damaging ER circuits. Pre-existing GI barrier and BBB alterations in EDD might also enhance this phenomenon. However, we would like to re-emphasize the importance of more extensive research on the topic, such as meta-analyses and works with greater generalization capabilities to draw statistically sound conclusions.

In BD, some studies demonstrated that proinflammatory mediators are associated with decreased brain volume in key areas responsible for ER. Others found a positive correlation between inflammation and alterations of networks involved in emotion processing and regulation. Moreover, although a certain level of chronic inflammation exists in most BD patients, certain subgroups seem more susceptible to peripheral (and possibly central) inflammation, especially those with a higher recurrence of manic episodes. If further work confirms that this ‘inflammatory subgroup’ includes patients with higher ED and that ED is mechanistically linked to inflammation, this could yield significant therapeutic implications, as further elaborated later in this section.

Finally, our review of patients with BPD showed that, while indirect, there are certain relationships between symptoms or mediators of ED and inflammation. For instance, both aggression and hostility (which can be ED symptoms) have been linked to alterations of specific inflammatory markers.

Overall, this narrative review did not enable us to identify specific peripheral or central inflammatory markers for these diseases, nor inflammatory markers exclusively associated with ED. It remains challenging to disentangle these results from the limited power of most reported studies and various factors that could contribute to conflicting results, such as variations in study design, sample characteristics, and other methodologies. Independent from the directionality of these findings, integrating clinical symptoms of ED with markers of inflammation, stress, and limbic connectivity could help characterise the physiopathology of BD, ADHD, and BPD. Assessing potential vulnerability markers for EDD remains crucial to identifying at-risk populations, especially in the context of these highly comorbid disorders. Peripheral blood inflammatory markers and cortisol levels can be measured routinely, and ER questionnaires can be administered easily in any healthcare facility. A joint evaluation of these measures could thus be integrated into screening procedures for subjects at risk, e.g*.*, individuals with a history of ELS or a familiarity with EDD.

Furthermore, unravelling the mechanisms linking ED and inflammation may provide a groundwork for new interventions to support the prevention and treatment of EDD. As supported by preliminary results [49,78,423], the use of adaptive ER strategies may positively impact peripheral inflammation and, possibly, modulate the sensitivity to stress. Vulnerable subjects can be trained to deploy effective ER strategies through psychosocial interventions, cognitive-behavioural therapy (CBT), or mindfulness [424] and mindfulness-based cognitive therapy, for instance. As far as EDD are concerned, one pilot study reported that mindfulness can improve ER in ADHD [425], although a recent meta-analysis has reported that results are not significant for this disorder, based on the few studies available [296]. A cross-sectional study also reported that BD patients had a lower disposition to mindfulness than HC [166], which was inversely correlated with depressive symptoms. Moreover, this association was largely explained by the DERS scores on the strategy subscale [166], which measures the belief that emotions can be controlled effectively during stressful events. These findings suggest that mindfulness could prove a viable intervention to improve ER in ADHD and BD patients. Interestingly, one recent study also proved that mindfulness could positively impact the FC of BD patients [426]. Mindfulness has additionally been suggested to modulate saliency and SMN processing [427], which are part of ER processing [268] and may be disrupted in EDD [250] (see also the BD section of this work). While there are no studies assessing the relationship between mindfulness, ED, and inflammation in EDD to our knowledge, previous work on healthy adults reported that mindfulness was associated with lower peripheral IL-6, and this association was partially explained by modifications in brain FC involving dlPFC [428]. Therefore, further investigation is warranted in this area, especially in the context of EDD.

To conclude, if the presumed connections between ED and inflammation presented in this work were confirmed, early interventions that target inflammation and ED in EDD could potentially mitigate the detrimental effects of these features on health and stress and ideally interrupt or at least modulate the vicious cycle summarized in Figure 2. Further characterization of the circuits linking ER strategies to inflammation might help validate this hypothesis and, eventually, identify context-appropriate strategies.

## Data Availability

Not applicable

## Open Access

Open access for this article was enabled by the participation of University of Geneva in an all-inclusive *Read & Publish* agreement with Portland Press and the Biochemical Society under a transformative agreement with Individual.

## Abbreviations

ACC: anterior cingulate cortex
AD: atopic dermatitis
BBB: blood–brain barrier
BD: bipolar disorder
BDNF: brain-derived neurotrophic factor
BMI: body mass index
cAMP: cyclic adenosine monophosphate
CBT: cognitive-behavioural therapy
CNS: central nervous system
CRP: C-reactive protein
CSF: cerebrospinal fluid
dACC: dorsal anterior cingulate cortex
DALY: disability-adjusted life years
DERS: Difficulties in Emotion Regulation Scale
dlPFC: dorsolateral prefrontal cortex
dmPFC: dorsomedial prefrontal cortex
dPFC: dorsal prefrontal cortex
ED: emotion dysregulation
ELS: early life stress
ER: emotion regulation
ERQ: Emotion Regulation Questionnaire
FC: functional connectivity
fMRI: functional magnetic resonance imaging
GWAS: genome-wide association studies
HC: healthy controls
HPA: hypothalamic–pituitary–adrenal
HsCRP: high-sensitivity C-reactive protein
IBS: irritable bowel syndrome
ICAM-1: intercellular adhesion molecule-1
IFG: inferior frontal gyrus
IFN-γ: interferon-γ
IL-10: interleukin-10
IL-18: interleukin-18
IL-1R: interleukin-1 receptor
IL-1β: interleukin-1β
IL-2: interleukin-2
IL-6: interleukin-6
IL-8: interleukin-8
IκBα: nuclear factor of kappa light polypeptide gene enhancer in B-cells inhibitor alpha
MCP-1: monocyte chemoattractant protein-1
MDD: major depressive disorder
MIDUS-II: Midlife in the United States-II
NAA: N-acetyl-aspartate
NAG: N-acetyl-aspartyl-glutamate
NFκB: nuclear factor kappa B
OFC: orbitofrontal cortex
p-PKCα: phosphorylated-PKC-α
PET: positron emission tomography
PFC: prefrontal cortex
PKC: protein kinase C
rsFC: resting-state functional connectivity
SAA: serum amyloid A
SCFA: short-chain fatty acid
SFG: superior frontal gyrus
sICAM-1: soluble intercellular adhesion molecule-1
sIL-2R: soluble IL-2 receptor
sIL-6R: soluble IL-6 receptor
SMN: somatomotor network
sTNF-R1: soluble tumour necrosis factor receptor-1
sVCAM-1: soluble vascular cell adhesion molecule-1
TAS: Toronto Alexithymia Scale
TNF-α: tumour necrosis factor-α
TSPO: 18-kDa translocator protein
vlPFC: ventrolateral prefrontal cortex
vmPFC: ventromedial prefrontal cortex
